# Supplementary material for: COMPASS for rapid combinatorial optimization of biochemical pathways based on artificial transcription factors
Source: Nat Commun. 2019 Jun 13;10:2615. doi: 10.1038/s41467-019-10224-x (PMC6565718; doi:10.1038/s41467-019-10224-x)
Supplement: Supplementary file 1 — Supplementary Information [file 41467_2019_10224_MOESM1_ESM.pdf]

Supplementary Figures

Color	ATF/BS	yEGFP geometric mean
	Pro$_{TDH3}$	819.3 ± 13.4
	NLS-JUB1-GAL4AD/2X	4043.4 ± 143.3[1]
	NLS-ATAF1-GAL4AD/2X	3612.5 ± 86.3[1]
	NLS-GAL4AD-ATAF1/2X	2568.1 ± 136.4[1]
	NLS-JUB1-EDLLAD-EDLLAD/4X	1890.8 ± 0.2[1]
	NLS-GAL4AD-ANAC102/4X	1616.2 ± 55.9[1]
	NLS-GAL4AD-GRF7/4X	1138.4 ± 32.4[1]
	ANAC102-NLS-VP64AD/4X	665.7 ± 14.9[1]
	NLS-DBD$_{JUB1}$-GAL4AD/2X	479.9 ± 25.4[1]
	NLS-GAL4AD-RAV1/4X	307.3 ± 18.7[1]

Supplementary Fig. 1. Plant-derived ATF/BSs used for COMPASS.

Nine different plant-derived ATFs and promoter pair combinations, providing weak (NLS-GAL4AD-RAV1/4X, NLS-DBD$_{JUB1}$-GAL4AD/2X, and ANAC102-NLS-VP64AD/4X; 300 - 700 AU), medium (NLS-GAL4AD-GRF7/4X, NLS-GAL4AD-ANAC102/4X and NLS-JUB1-EDLLAD-EDLLAD/4X; 1,100 - 1,900 AU) and strong (NLS-GAL4AD-ATAF1/2X, NLS-ATAF1-GAL4AD/2X and NLS-JUB1-GAL4AD/2X; 2,500 - 4,000 AU) transcriptional outputs, were selected from the previously characterized library of plant-derived ATF/BS integrated into the *ura3-52* locus[1]. The constitutive yeast *TDH3* promoter integrated into the same locus (819.3 ± 13.4 AU) was used as a control. AU, arbitrary units, determined using EGFP as reporter[1]. ´2X´ and ´4X´ indicate the number of bindings sites implemented for the ATFs within the minimal *CYC1* promoters. The color code is used for presenting the results in Figs. 5h and 6b, and Supplementary Figs. 15d, 16c and 17d.

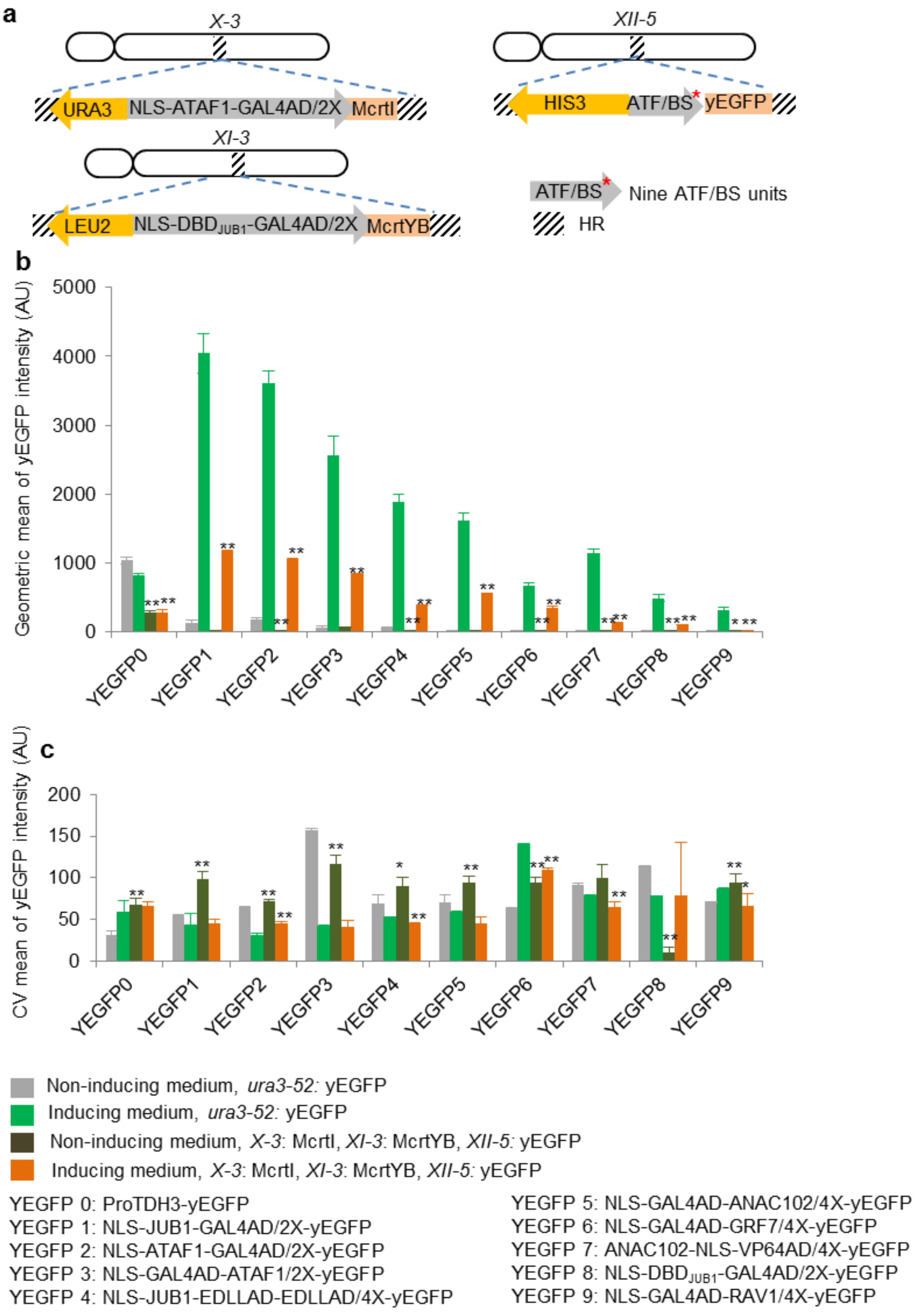

Supplementary Fig. 2. Position effects of plant-derived regulators on yEGFP level.

(a) CRISPR/Cas9-mediated integration of the *yEGFP*, *McrtYB* and *McrtI* modules into three *sites of the yeast genome*. The *yEGFP*-, *McrtYB*- and *McrtI*-donors were integrated into locus *XII-5*, *XI-3*, and *X-3*, respectively. Donors contain regulator-CDS modules and yeast auxotrophic markers flanked by HRs for integration into the desired yeast genomic sites. Expression of *McrtYB* and *McrtI* in *McrtYB* and *McrtI* donors is under the control of NLS-ATAF1-GAL4AD-2X and NLS-DBD$_{JUB1}$-GAL4AD-4X, respectively. Expression of yEGFP in

ATF/BS-yEGFP donors is under the control of nine different plant-derived ATF/BSs. Expression of yEGFP under the control of the constitutive yeast *TDH3* promoter served as positive control. Selection on SC-Ura/-Leu/-His medium allows screening for successfully integrated cassettes. Grey arrows, nine ATF/BS units. Black/white-striped squares, homology regions. For simplicity, the IPTG-inducible promoters upstream of the ATF/BS, terminator fragments, the HRs needed for cloning, and cleavage sites flanking the genes are not included in the figure. The geometric mean **(b)** and CV mean **(c)** of yEGFP intensity for plant-derived ATF/BS. yEGFP output was tested in strains YEGFP1 - YEGFP9 in the absence and presence of inducer. For comparison, we also included our previously reported data obtained for plant-derived ATF/BS-yEGFP modules integrated in locus *ura3-52*[1] (adapted with permission from*:* Naseri *et al*. (2017) Plant-derived transcription factors for orthologous regulation of gene expression in the yeast *Saccharomyces cerevisiae.* ACS Synthetic Biology 6(9), 1742-1756; DOI: 10.1021/acssynbio.7b00094. Copyright 2017, American Chemical Society). Data are means ± SD of the fluorescence intensity per cell, obtained from three cultures, each derived from an independent yeast colony and determined in three technical replicates (n = 9). AU, arbitrary units. Asterisks indicate statistically significant differences from the strain expressing the respective plant-derived ATF/BS integrated into the *ura3-52* locus (Student´s *t*-test; * $p < 0.05$; ** $p < 0.01$). Full data of Supplementary Figure 2b - c are given in Supplementary Data 2a - b.

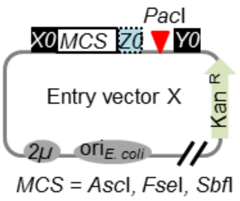

Supplementary Fig. 3. Design of Entry vector X.

The backbone contains the *E. coli pUC19* replication origin, the kanamycin resistance gene *nptII*, the yeast *2μ* replication origin, an *MCS* flanked by *X0* and *Z0* sequences, and a *Pac*I site flanked by *Z0* and *Y0* sequences. The vector is used to establish ATF/BS units (ATF and BS) within the *MCS*, and to assemble CDS units (CDS, yeast terminator, and the promoter of an *E. coli* selection marker) at the *Pac*I site. *X0*: upstream (left) HR of the vector that provides homology to the forward primer of the first part of the ATF/BS unit (primer ATF_for). *Z0*: the last 30 bp of the minimal *CYC1* promoter. *Y0:* downstream (right) HR of the vector that provides homology to the reverse primers of the last part of the CDS unit (promoter of an *E. coli* selection marker).

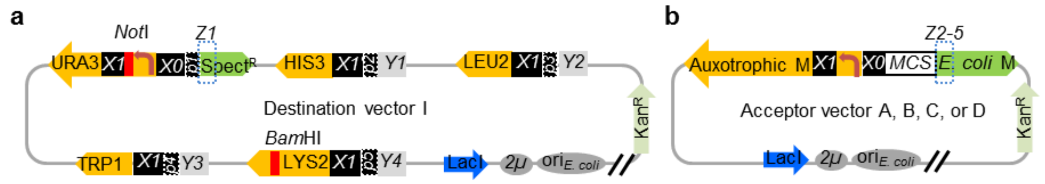

M: Marker
MCS: AscI, FseI, SbfI
p1: *Sal*I/*Eco*RI, *p2*: *I-Ceu*I/*Sbf*I, *p3*: *I-Sce*I/*Fse*I, *p4*: *PI-Psp*I/*Sfi*I, *p5*: *Asc*I

Supplementary Fig. 4. Design of Set 1 vectors.

All vectors contain the *E. coli pUC19* replication origin, the kanamycin resistance gene *nptII*, and the yeast 2μ replication origin. **(a)** Destination vector I has cloning sites *p1* (*Sal*I/*Eco*RI), *p2* (*I-Ceu*I/*Sbf*I), *p3* (*I-Sce*I/*Fse*I), *p4* (*PI-Psp*I/*Sfi*I), and *p5* (*AscI*). The *p1* site is flanked by *X0* and *Z1*, where a *URA3* marker is placed upstream (left) of *X0. X0* gives a HR to the Entry vector X, while *Z1* gives a HR to the 3' end of *E. coli* markers. The *p2*, *p3*, *p4*, and *p5* sites are flanked upstream (left) by *X1* and downstream (right) by *Y1*, *Y2*, *Y3*, and *Y4*, respectively. The CDSs and terminators of *HIS3*, *LEU2*, *TRP1* and *LYS2* are fused upstream (right) to *X1* of *p2*, *p3*, *p4*, and *p5*, respectively. *X1* provides an HR to the forward primer amplifying the ATF/BS-CDS module (primer X1_for) in the Level 1 assembly procedure, while *Y1*, *Y2*, *Y3*, and *Y4* provide HRs to the last 30 bp of the terminator of Amp^R, Cm^R, TCS^R, and Gen^R, respectively. Destination vector I is equipped with *Not*I and *Bam*HI sites allowing integration of the plasmid into *URA3* and *LYS2* loci of the yeast genome, respectively. **(b)** Acceptor vectors with auxotrophic markers. Acceptor vector A, B, C, or D has an *MCS* flanked by *X0* and *Z2, Z3, Z4,* or *Z5*, respectively, providing HRs to the appropriate *E. coli* markers. In each Acceptor vector, a yeast auxotrophic marker (A: *HIS3*; B: *LEU2*; C: *TRP1; and* D: *LYS2*) is placed upstream (left) of *X0*. The HRs *X0, X1* and *Z1 - Z5* are explained in footnote to Supplementary Data 4.

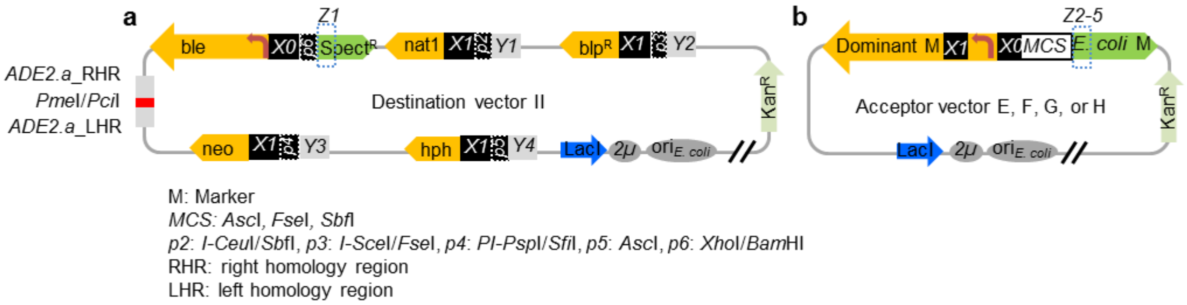

Supplementary Fig. 5. Design of Set 2 vectors.

(a) Destination vector II is similar to Destination vector I (see Supplementary Fig. 4a), except for the following: (1) site *p1* is replaced by site *p6* (*Xho*I/*Bam*HI), (2) the URA3 encoding fragment is replaced by a functional *Ble* auxotrophic marker, and (3) the CDSs and terminators of *HIS3*, *LEU2*, *TRP1* and *LYS2* are replaced by the CDSs and terminators of nat1, blp^R, neo, and hph, respectively. Destination vector II is equipped with *Pme*I and *Pci*I restriction sites flanked by HRs to allow integration into the *ADE2.a* locus of yeast. **(b)** Acceptor vectors with dominant markers. Acceptor vectors E, F, G, or H have an *MCS* flanked by an *X0* sequence in the 5' region and *Z2, Z3, Z4,* or *Z5* sequences in the 3' region. *Z2, Z3, Z4,* and *Z5* sequences represent the first 30 bp of the *Amp^R* (E), *Cm^R* (F), *TCS^R* (G) or *Gen^R* (H) CDS, respectively, and provide HRs to the appropriate *E. coli* selection markers present in the CDS units in Level 0. In each Acceptor vector a functional yeast dominant selection marker (E: nat1, F: blp^R, G: neo, and H: hph) is placed upstream (left) of the *X0* sequence. All Destination and Acceptor vectors harbor the *E. coli pUC19* replication origin, the kanamycin resistance gene *nptII*, the yeast *2μ* replication origin, and a yeast selection marker.

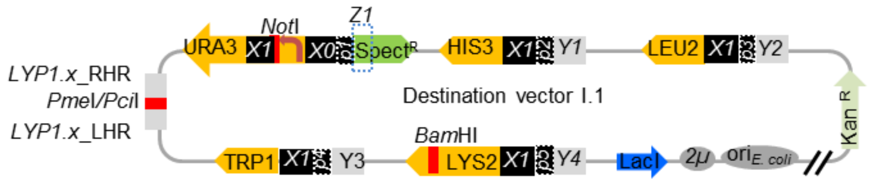

MCS = *Asc*I, *Fse*I, *Sbf*I
M: Marker
p1: *Sal*I/*Eco*RI, *p2*: *I-Ceu*I/*Sbf*I, *p3*: *I-Sce*I/*Fse*I, *p4*: *PI-Psp*I/*Sfi*I, *p5*: *Asc*I
RHR: right homology region
LHR: left homology region

Supplementary Fig. 6. Design of Destination vector I.1.

Destination vector I.1 contains the *E. coli pUC19* replication origin, the kanamycin resistance gene *nptII*, the yeast *2μ* replication origin, and a yeast selection marker. Moreover, it has cloning sites *p1* (*Sal*I/*Eco*RI), *p2* (*I-Ceu*I/*Sbf*I), *p3* (*I-Sce*I/*Fse*I), *p4* (*PI-Psp*I/*Sfi*I), and *p5* (*AscI*). Site *p1* is flanked by *X0* and *Z1*, and the *URA3* auxotrophic selection marker is located upstream (left) of *X0. X0* provides a HR to the Entry vector X, while *Z1* overlaps to the first 30 bp of the Spect^R CDS present in the backbone and provides HRs to the appropriate *E. coli* selection markers present in the CDS units in Level 0. Sites *p2*, *p3*, *p4*, and *p5* are flanked by *X1* in the 5´ region, and by *Y1*, *Y2*, *Y3*, and *Y4* in the 3´ region. The CDSs and terminators of *HIS3*, *LEU2*, *TRP1* and *LYS2* fused to *X1* are located upstream (right) of sites *p2*, *p3*, *p4*, and *p5*, respectively. *X1* provides an HR to the forward primer amplifying the ATF/BS-CDS module (primer X1_for) in the Level 1 assembly procedure, while the Level 2 *Y1*, *Y2*, *Y3*, and *Y4* provide HRs to the last 30 bp of the terminator sequences of *Amp*^{R} (primer Y2_rev), *Cm*^{R} (primer Y3_rev), *TCS*^{R} (primer Y3_rev), and *Gen*^{R} (primer Y4_rev), respectively. To integrate the pathways assembled in Destination vector I.1 into the genome, it is equipped with *Pme*I (and *Pci*I) recognition sequence flanked by HRs for integration into the *LYP1.x* locus[2].

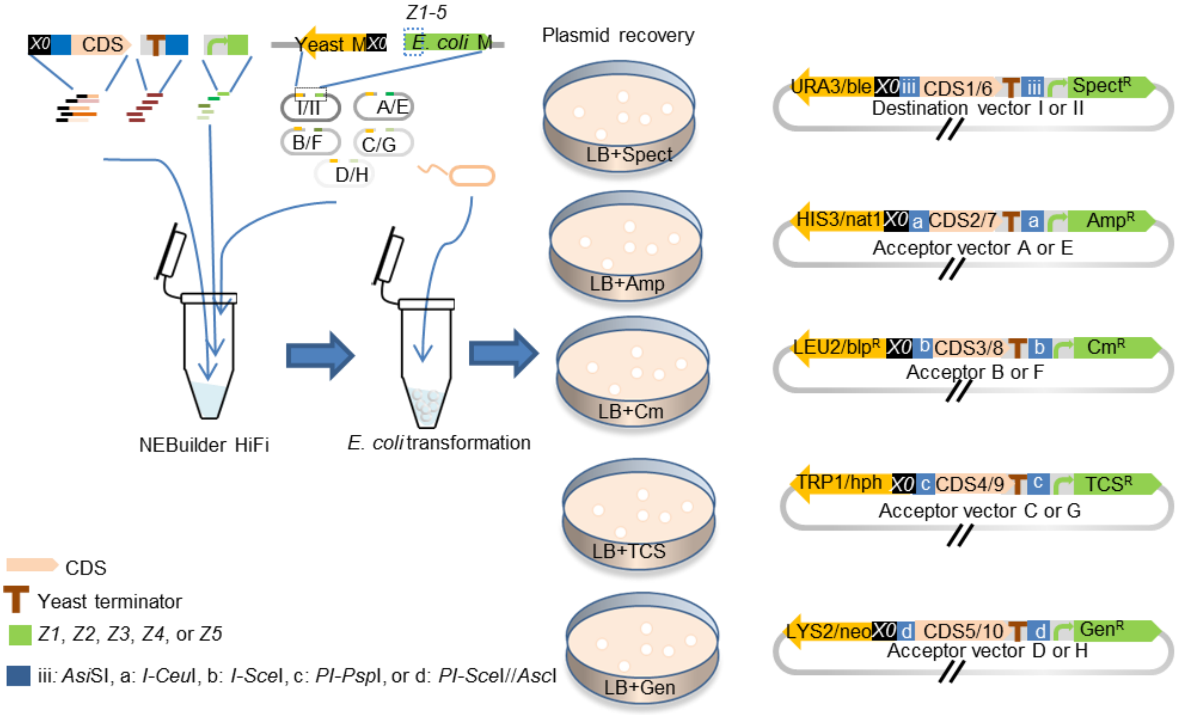

Supplementary Fig. 7. Combinatorial assembly of pathway gene units.
Equal amounts of five freely selected CDSs, equal amounts of five yeast terminators, equal amounts of five promoters of *E. coli* selection markers (*Spect*^{R}*, Amp*^{R}, *Cm*^{R}, *TCS*^{R}, and *Gen*^{R}), and equal amounts of digested vectors from Level 1 (*Sal*I/*Eco*RI-digested Destination vector I or *Xho*I/*Bam*HI- digested Destination vector II, *Fse*I/*Asc*I-digested Acceptor vectors A or E, B or F, C or G, and D or H) are mixed in a single tube for overlap-based cloning. The upstream (left) HR of the CDS parts overlap with the *X0* sequence of the plasmid backbone and rare RE cleavage sites (Destination vectors I or II: iii, Acceptor vectors A or E: a, B or F: b, C or G: c, and E or F: d), compatible to the next level vectors, are introduced before the gene´s translation start codon through primer sequences. Moreover, the upstream HR of the promoter of the antibiotic resistance gene is defined by the yeast terminator; the same rare RE recognition site downstream of the terminator is introduced, while the downstream (right) HR of the *E. coli* promoter is defined based on *Z* sequences (*Z1*: *Spect*^{R}, *Z2*: *Amp*^{R}, *Z3*: *Cm*^{R}, *Z4*: *TCS*^{R}, or *Z5*: *Gen*^{R}). The positive selection of truncated plasmid markers is rendered active by providing promoter sequences during the assembly process. Therefore, Destination vector I-CDS1 or II-CDS6, Acceptor vectors A-CDS2 or E-CDS7, B-CDS3 or F-CDS8, C-CDS4 or G-CDS9, and D-CDS5 or H-CDS10 are generated. The HRs *X0* and *Z1 to Z5* are explained in footnote to Supplementary Data 4. Light-orange arrows, CDS. Brown ´T´, yeast terminator. Green squares, *Z1*, *Z2*, *Z3*, *Z4*, or *Z5*. Blue squares, iii: *Asi*SI, a: *I-Ceu*I, b: *I-Sce*I, c: *PI-Psp*I, d: *PI-Sce*I/*Asc*I.

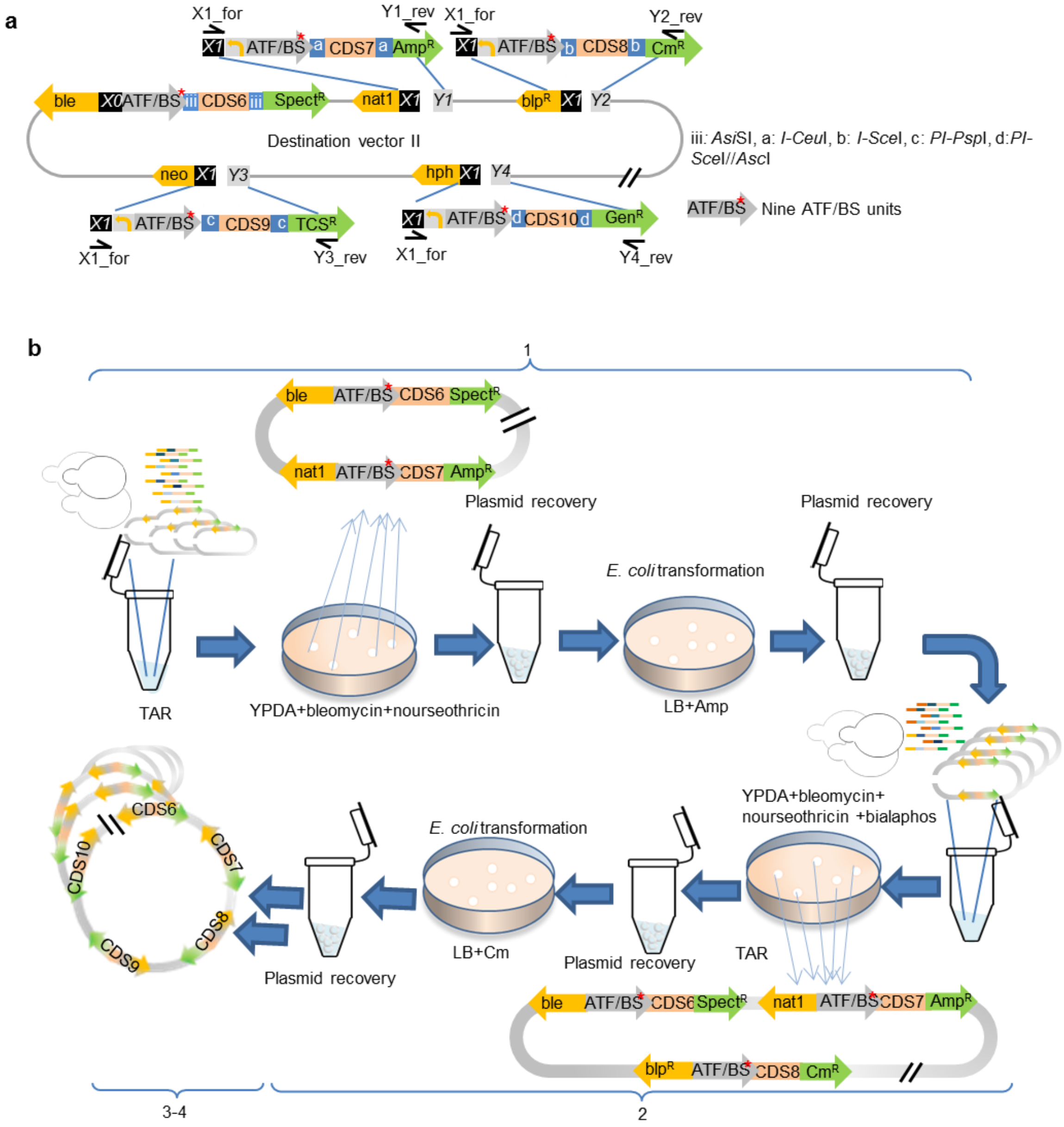

Supplementary Fig. 8. Combinatorial cloning of pathway modules.

Libraries of PCR-amplified *Pro_{nat1}-ATF/BS-CDS7-Pro_{AmpR}-AmpR-Ter_{AmpR}* (primers X1_for/Z2_rev, on Acceptor vector E), *Pro_{blpR}-ATF/BS-CDS8-Pro_{CmR}-CmR-Ter_{CmR}* (primers X1_for/Z3_rev, on Acceptor vector F), *Pro_{neo}-ATF/BS-CDS9-Pro_{TCSR}-TCSR-Ter_{TCSR}* (primers X1_for/Z4_rev, on Acceptor vector G), and *Pro_{hph}-ATF/BS-CDS10-Pro_{GenR}-GenR-Ter_{GenR}* (primers X1_for/Z5_rev, on Acceptor vector H) modules are successively assembled in sites *p2*, *p3*, *p4*, and *p5,* respectively, starting from the Destination vectors I-CDS6 library (see Supplementary Fig. 4) in four rounds of combinatorial cloning. **(b)** The COMPASS workflow for combinatorial assembly of pathway genes in Destination vector II. The mixed ATF/BS-CDS7 modules are assembled in site *p2* of Destination vectors II-CDS6 using TAR. Yeast cells with successful assemblies grow on YPDA medium containing bleomycin and nourseothricin. Cells are scraped from the plates and the plasmid library is extracted to

obtain a pool of all randomized members. The plasmid library is transformed into *E. coli*, and cells are grown on LB plates containing ampicillin. Next, cells are scraped from the plates and the plasmid library is extracted (1). The ATF/BS-CDS8 modules are assembled in site *p3* of Destination vectors I-CDS6-CDS7 using TAR. Yeast cells with successful assemblies grow on YPDA medium containing bleomycin, nourseothricin and bialaphos (2). Using TAR, the libraries of ATF/BS-CDS9 and ATF/BS-CDS10 modules are cloned in sites *p4* and *p5*, respectively, in two further rounds of combinatorial clonings (3 - 4). Grey arrows, nine ATF/BS units. Blue squares; iii: *Asi*SI, a: *I-Ceu*I, b: *I-Sce*I, c: *PI-Psp*I, and d: *PI-Sce*I/*Asc*I. For simplicity, IPTG-inducible promoters and terminators are not included in the figure. The HRs *X0*, *X1*, and *Y1-Y4* are explained in footnote to Supplementary Data 4.

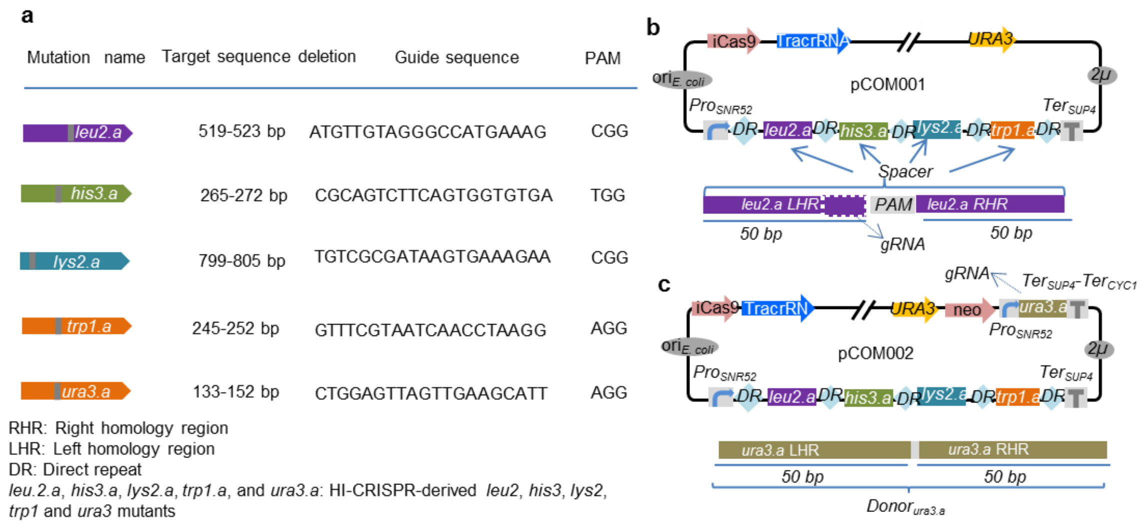

Supplementary Fig. 9. CRISPR-Cas9-mediated disruption of auxotrophic markers.

(a) CRISPR-Cas9 target site selection for frame-shift mutations of *LEU2*, *HIS3*, *LYS2*, *TRP1*, and *URA3* genes according to Bao *et al.* (2015)[2]. The relative positions of target sites within each gene and the guide RNA and PAM sequences of each site are shown. **(b)** Schematic presentation of quadruple auxotrophic marker gene disruption using the one-plasmid HI-CRISPR system[2]. Plasmid pCOM001 contains donor sequences for *LEU2*, *HIS3*, *LYS2*, and *TRP1* gene disruption. For each gene disruption, a 100-bp donor sequence containing a gRNA targeting a 20-bp target and the PAM sequence was designed to harbor two 50-bp HRs flanking the Cas9 cleavage site. **(c)** Schematic presentation of quintuple auxotrophic marker gene disruption. Plasmid pCOM002 is derived from pCOM001. It contains a functional yeast *neo* (G418) resistance gene, Pro_{SNR52} and Ter_{SUP4} (fused to yeast Ter_{CYC1}) to control the transcription of the 20-bp gRNA to disrupt *URA3* gene expression, in addition to *LEU2*, *HIS3*, *LYS2*, and *TRP1*. Thereby, transformation of pCOM002 together with the 100-bp *ura3.a* donor into the yeast host cell results in quintuple auxotrophic marker gene disruption.

a

Mutation name	Target sequence deletion	Guide sequence	PAM
ble.a	169-176 bp	AGCAGCTGAACCAACTCGCG	AGG
nat1.a	158-165 bp	GGGAACACCTTGGTCAGGGG	GGG
blpR.a	109-116 bp	CGACGAGCCAGGGGTAGCGG	AGG
neo.a	161-168 bp	AAAGGTAGCGTTGCCAATGA	CGG
hph.a	307-314 bp	CGGGCAGTTCGGTTTCAGGC	AGG

b

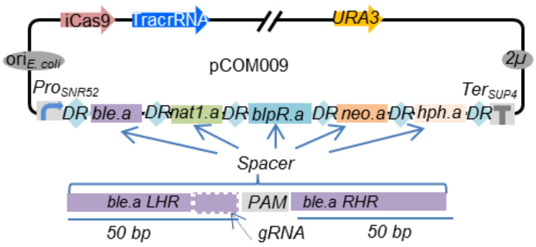

RHR: Right homology region
LHR: Left homology region
DR: Direct repeat
ble.a, *nat1.a*, *blpR.a*, *neo.a*, and *hph.a*: HI-CRISPR-derived *ble*, *nat1*, *blpR*, and *hph* mutants

Supplementary Fig. 10. CRISPR-Cas9-mediated disruption of dominant markers.

(a) CRISPR-Cas9 target site selection for frame-shift mutations of *ble*, *nat1*, *blpR*, *neo*, and *hph* genes according to Bao *et al.* (2015)[2]. The target site positions within each gene, and the guide RNA and PAM sequences are shown. **(b)** Schematic presentation of quintuple dominant marker gene disruption using HI-CRISPR[2]. Plasmid pCOM009 harbors donor sequences for the *ble*, *nat1*, *blp*^{R}, *neo*, and *hph* genes. For each gene disruption, a 100-bp donor fragment encompassing a gRNA sequence (addressing a 20-bp target), the PAM, and two 50-bp HRs flanking the Cas9 cleavage site was generated.

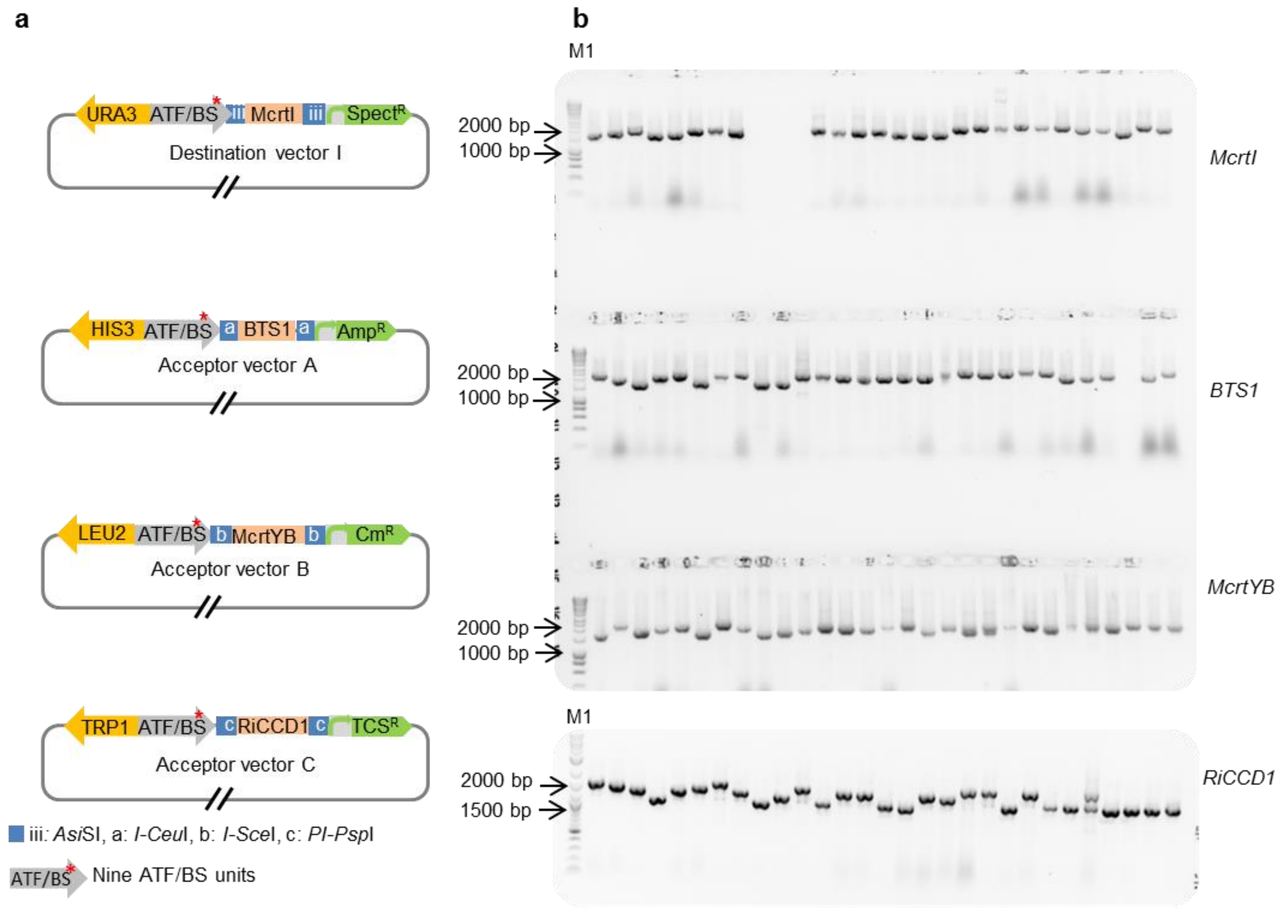

Supplementary Fig. 11. Construction of the β-ionone pathway library.

(a) Schematic overview of modules of ATF/BS library and pathway genes. Nine ATF/BS control units and β-carotene (*McrtI*, *BTS1*, *McrtYB*), and *RiCCD1* CDS units (RiCCD1 converts β-carotene to β-ionone) were assembled in Destination vector I, Acceptor vectors A, B, and C, respectively. **(b)** Agarose gel electrophoresis for the identification of constructed ATF/BS and β-ionone CDS modules. The nine ATF/BS units were assembled upstream of β-ionone CDSs in a single cloning tube in a combinatorial manner. By plating the transformed cells onto LB agar medium containing either spectinomycin, ampicillin, chloramphenicol, or triclosan, libraries of Destination vector I, Accepter vector A, B, and C, differing in ATF/BS units upstream of *McrtI*, *BTS1*, *McrtYB,* and *RiCCD1* modules, were generated. The verification of transformation was done using colony PCR followed by sequencing (see Methods). Gray arrows, nine ATF/BS units. Blue squares; iii: *AsiS*I, a: *I-Ceu*I, b: *I-Sce*I, and c: *PI-Psp*I. For simplicity, the IPTG-inducible promoters upstream of the ATF/BS, terminator fragments, and HRs needed for cloning are not included in the figure. M1: HyperLadder 1 kb (Bioline).

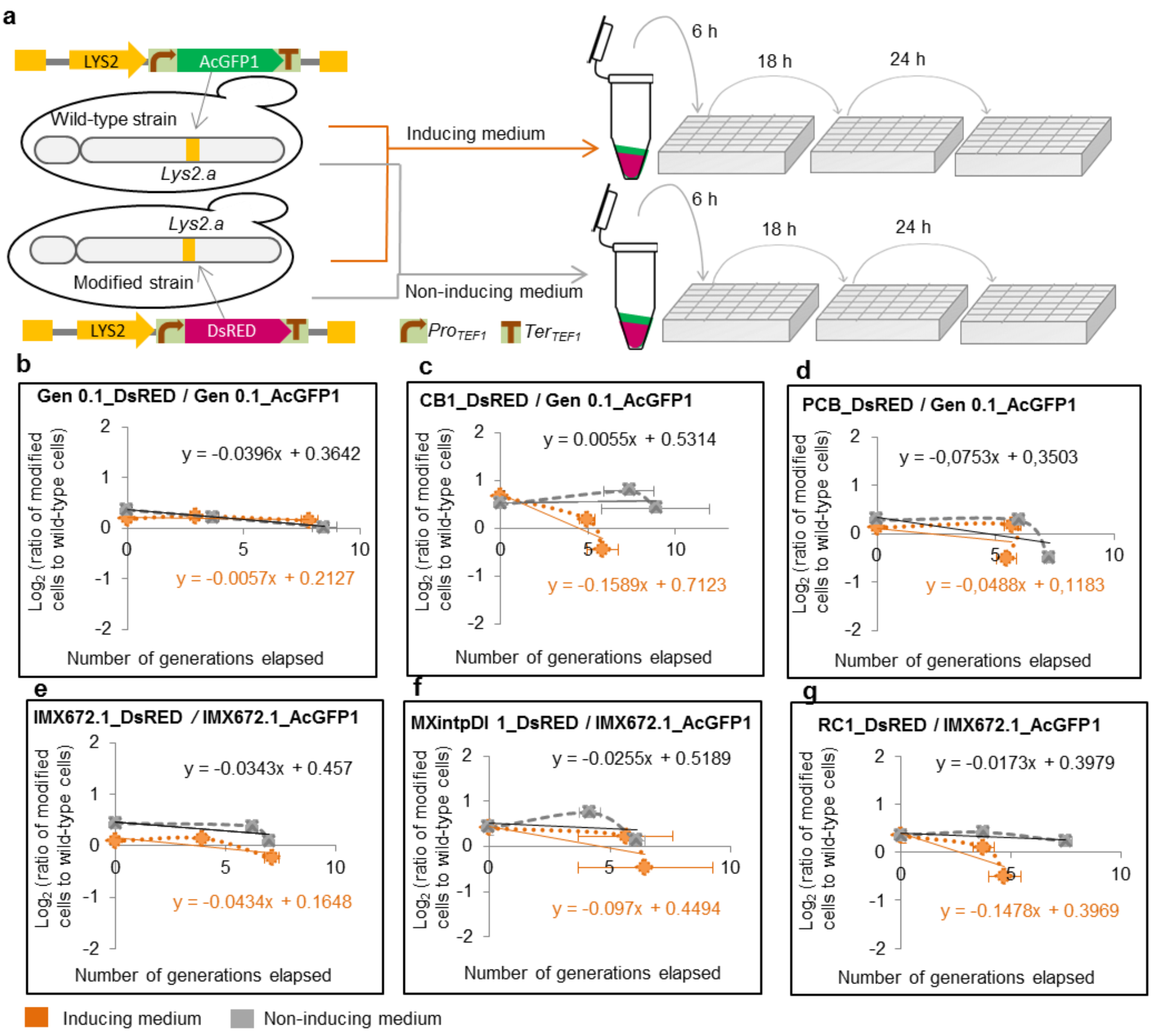

Supplementary Fig. 12. High-precision flow cytometry for growth analysis.
(a) Schematic overview of the assay. DsRED- and AcGFP1-encoding plasmids harboring the *LYS2* gene as selection marker and either DsRED or AcGFP1 coding sequences under the control of the *TEF2* promoter are integrated into the yeast *lys2.a* site of the strain of interest and the corresponding wild-type strain. The AcGFP1-expressing wild-type strain is co-cultured with a DsRED-expressing strain at a cell ratio of 2 : 8, in a single vessel with cultures maintained by serial dilution over a 48-h period. The relative abundance of each strain is monitored over time by flow cytometry. Brown bent arrow, yeast *TEF1* promoter. Brown ´T´, yeast *TEF1* terminator. Relative growth rates of yeast strains **(b)** Gen 0.1, **(c)** IMX672.1, **(d)** PCB, **(e)** CB1, **(f)** MXintpDI 1, and **(g)** RC1. The orange and grey lines indicate the rate of DsRED-labelled strain depletion over time in inducing and non-inducing medium, respectively. Data are means ± SD of the logarithm of the ratio of modified cells to wild-type cells, obtained from three cultures, each derived from an independent yeast colony and determined in three technical replicates (n = 9). Full data of Supplementary Figures 12b – g are given in Supplementary Data 8a - f.

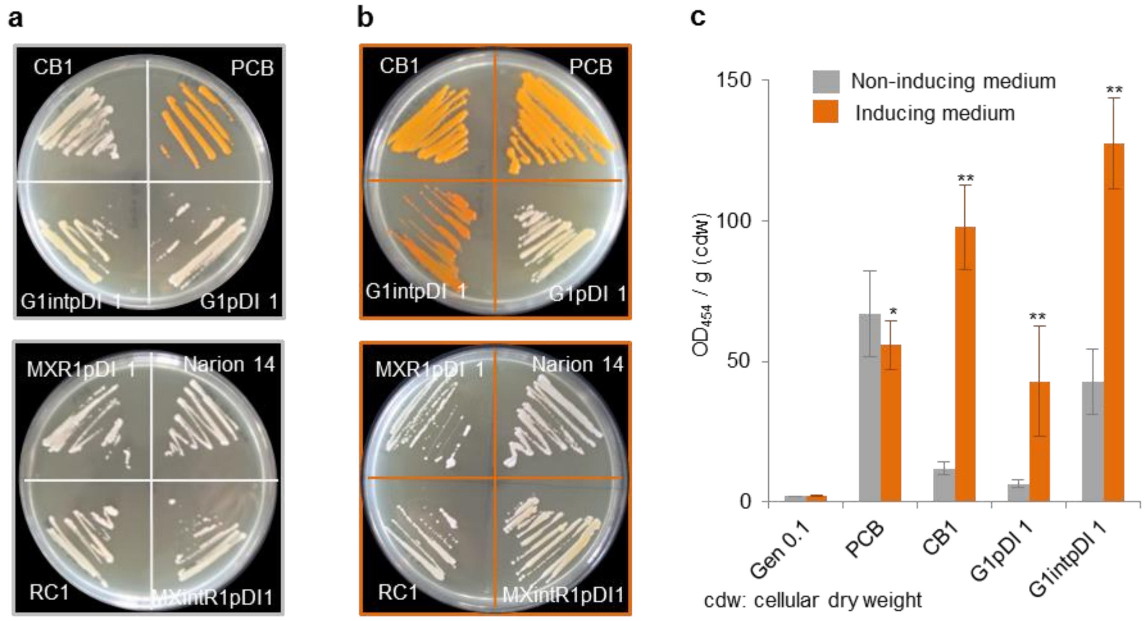

Supplementary Fig. 13. Controllability of plant-derived regulators in COMPASS strains. (a) Carotenoid production in the absence and **(b)** presence of inducer**.** The best yeast producers of each of the three COMPASS approaches (see Fig. 5, Supplementary Fig.15-17), *i.e.*, CB1, G1pDI 1, G1intpDI 1 (β-carotene), RC1, MXR1pDI, MXintR1pDI1 (β-ionone) and Narion 14 (β-ionone), were plated on non-inducing and inducing SC medium with appropriate selection markers. Strain PCB, expressing β-carotene pathway genes under the control of the constitutive yeast *TDH3* promoter, was used as a control. **(c)** β-Carotene quantification in non-inducing and inducing media using the method reported by Lian *et al*. (2017)[3] for Gen 0.1, CB1, G1pDI 1, and G1intpDI 1. The β-carotene absorbance at 454 nm was measured and normalized to the cell density; the data are means ± SD of nine cultures derived from independent colonies of each strain (n = 9). Asterisks indicate statistically significant difference from non-inducing medium (Student´s *t*-test; * $p < 0.05$; ** $p < 0.01$). DW, cell dry weight. Full data of Supplementary Figure 13c are given in Supplementary Data 9.

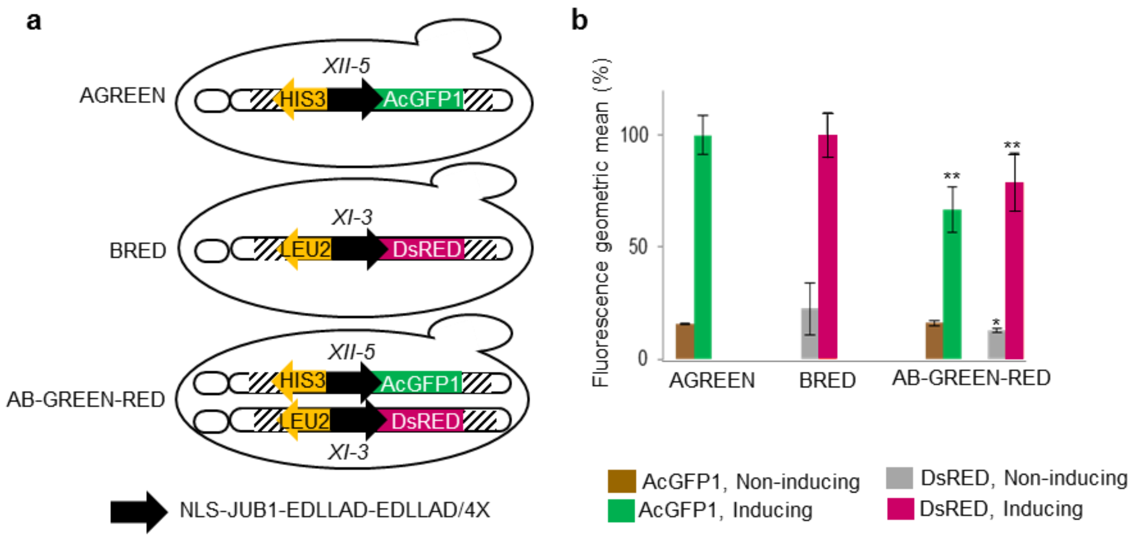

Supplementary Fig. 14. Effect of the ATF NLS-JUB1-EDLLAD-EDLLAD on transcriptional output towards JUB1 binding sites.

(a) Schematic overview of CRISPR/Cas9-mediated integration of regulator and fluorescent protein-encoding DNA modules into the yeast genome. The AVA-GREEN- and AVB-RED-donors driving expression of AcGFP1 and DsRED reporters, respectively, can be integrated into the *XII-5* and *XI-3* loci. Each donor contains the NLS-JUB1-EDLLAD-EDLLAD/4X regulatory unit (black arrow) upstream of the fluorescent protein CDS unit, and a yeast dominant marker. Moreover, each donor is flanked by HRs for integration into the desired locus. Selection of yeast strains on SC-Ura/-Leu/-His/-Trp medium allows screening for gene cassettes successfully integrated into the yeast genome. For simplicity, the IPTG-inducible promoters upstream of the ATF/BS, terminator fragments, the HRs needed for cloning, and cleavage sites flanking fluorescent protein encoding genes are not included in the figure. **(b)** Integration of donor DNA encoding the reporter protein into the genomic locus. The AcGFP1 donor was integrated into the *XII-5* locus of yeast strain IMX672.1 to generate strain AGREEN. The DsRED donor was integrated into the *XI-3* locus of the IMX672.1 genome to generate strain BRED. The AcGFP1 and DsRED donors were integrated into the sites *XII-5* and *XI-3* of the IMX672.1 genome, respectively, to generate strain AB-GREEN-RED. The AcGFP1 and DsRED outputs were tested in the absence and presence of inducer using flow cytometry. Data are geometric means ± SD of the fluorescence intensity per cell obtained from three cultures, each derived from an independent yeast colony and determined in three technical replicates (n = 9). Asterisks indicate statistically significant differences from the strain expressing the respective fluorescent protein (*i.e.*, AcGFP1-expression in strain AB-GREEN-RED *vs.* AGREEN, and DsRED-expression in strain AB-GREEN-RED *vs.* BRED; Student´s *t*-test; * $p < 0.05$; ** $p < 0.01$). AU, arbitrary units. Full data of Supplementary Figure 14b are given in Supplementary Data 11.

.

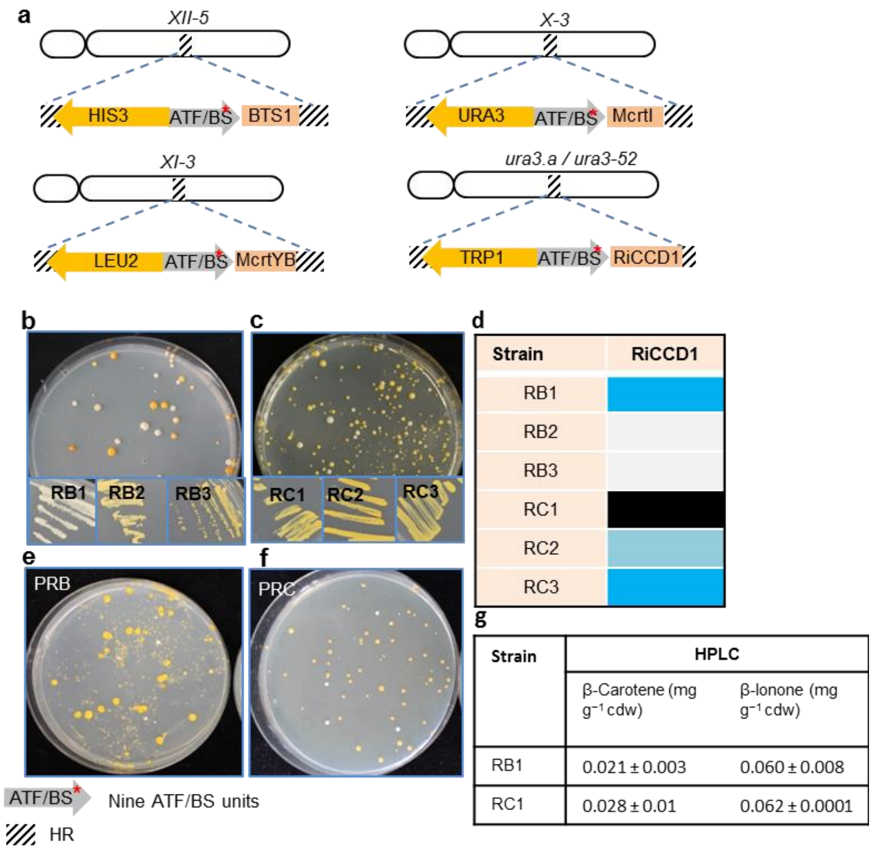

Supplementary Fig. 15. Multi-locus integration of β-ionone pathway genes.

(a) CRISPR/Cas9-mediated integration of the four β-ionone pathway gene donors into four different *loci of the yeast genome*. The *McrtI*-, *McrtYB*-, *BTS1*- and *RiCCD1*-donors driving β-ionone expression were integrated into the *X-3*, *XI-3*, *XII-5* and *ura3-52* loci. Donors contain the ATF/BS-CDS modules and the yeast dominant markers that are flanked by HRs for integration into the desired loci. Selection on SC-Ura/-Leu/-His/-Trp medium allows screening for successfully integrated cassettes. Grey arrows, nine ATF/BS units. Black/white-striped squares, homology regions. For simplicity, the IPTG-inducible promoters upstream of the ATF/BS, terminator fragments, the HRs needed for cloning, and cleavage sites flanking pathway genes are not included in the figure. **(b)** The library of donors containing the ATF/BS-RiCCD1 modules was integrated into the *ura3-52* locus of two strains producing high levels of β–carotene, namely strain CB1 in the Gen 0.1 background to generate RB variants and **(c)** strain CC1 in the IMX672.1 background to generate RC variants. **(d)** Sequencing results for ATF/BS units upstream of the *RiCCD1* CDS from three different colonies producing light color in either CB1 (RB1, RB2, and RB3) or CC1 (RC1, RC2, and RC3) backgrounds. The color code is given in Supplementary Fig. 1. **(e)** Modules containing the β-ionone CDSs under the control of the constitutive yeast *TDH3* promoter in strain Gen

0.1 to generate PRB, or **(f)** strain IMX672.1 to generate PRC. **(g)** HPLC analysis of strains RB1 and RC1.

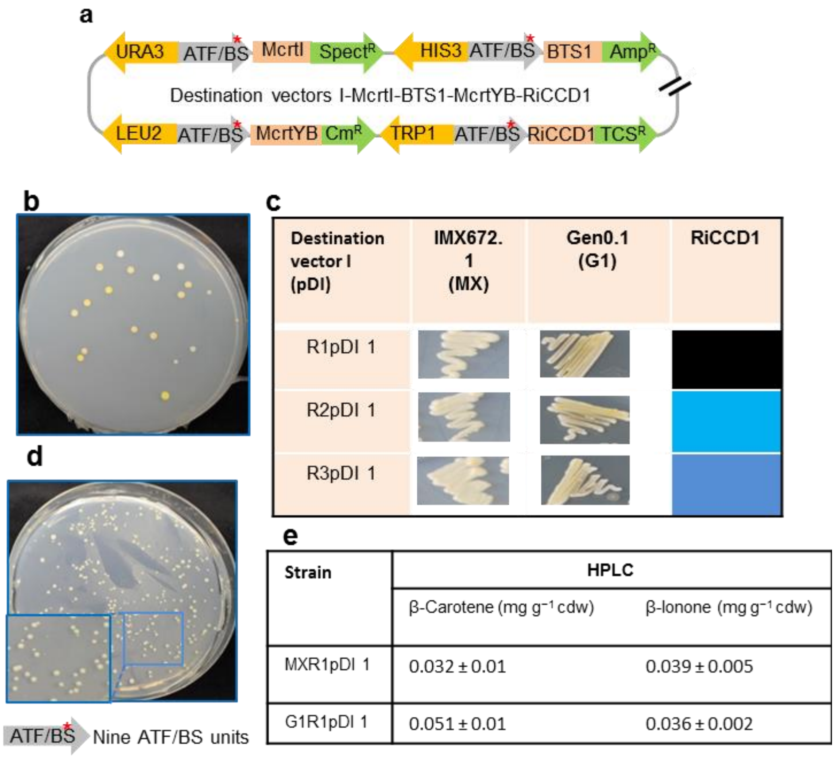

Supplementary Fig. 16. β-Ionone pathway library in Destination vector I.

(a) Schematic overview of assembly of the ATF/BS and β-ionone pathway library in Destination vector I. The libraries of the *McrtI*-, *McrtYB*-, *BTS1,* and *RiCCD1* modules, under the control of ATF/BS regulator units, were assembled in Destination vector I. The ATF/BS-*McrtI*, -*McrtYB*-, -*BTS1*, -*RiCCD1* modules are flanked by yeast auxotrophic markers *URA3*, *HIS3*, *LEU2*, and *TRP1* at the 5' end, while *E. coli* selection markers Spect^R, Amp^R, Cm^R, and TCS^R are placed at the 3' end. Selection on SC-Ura/-Leu/-His/-Trp medium or LB medium containing spectinomycin, ampicillin, chloramphenicol, and triclosan allows screening for successfully assembled constructs. Grey arrows, nine ATF/BS units. For simplicity, the IPTG-inducible promoters upstream of the ATF/BS, terminator fragments, the HRs needed for cloning, the cleavage sites flanking the pathway genes and terminators are not included in the figure. **(b)** Transformation of the library of modules containing the *RiCCD1* CDSs under the control of the nine ATF/BS regulators into strain Gen 0.1. **(c)** Library of plasmids extracted from yeast and transformed into *E. coli* followed by sequencing. Three sequenced plasmids (R1pDI 1, R2pDI 1, and R3pDI 1) were retransformed into the Gen 0.1 and IMX672.1 backgrounds. The color code indicating the ATF/BS units assembled upstream of the CDS coding sequences is given in Supplementary Fig. 1. **(d)** Plasmid pIONONE-PTDH3 containing the β-ionone CDSs under the control of the constitutive yeast *TDH3* promoter was

transformed into strain Gen 0.1. **(e)** HPLC analysis of MXR1pDI 1 and G1R1pDI 1 producing the shallow yellow color.

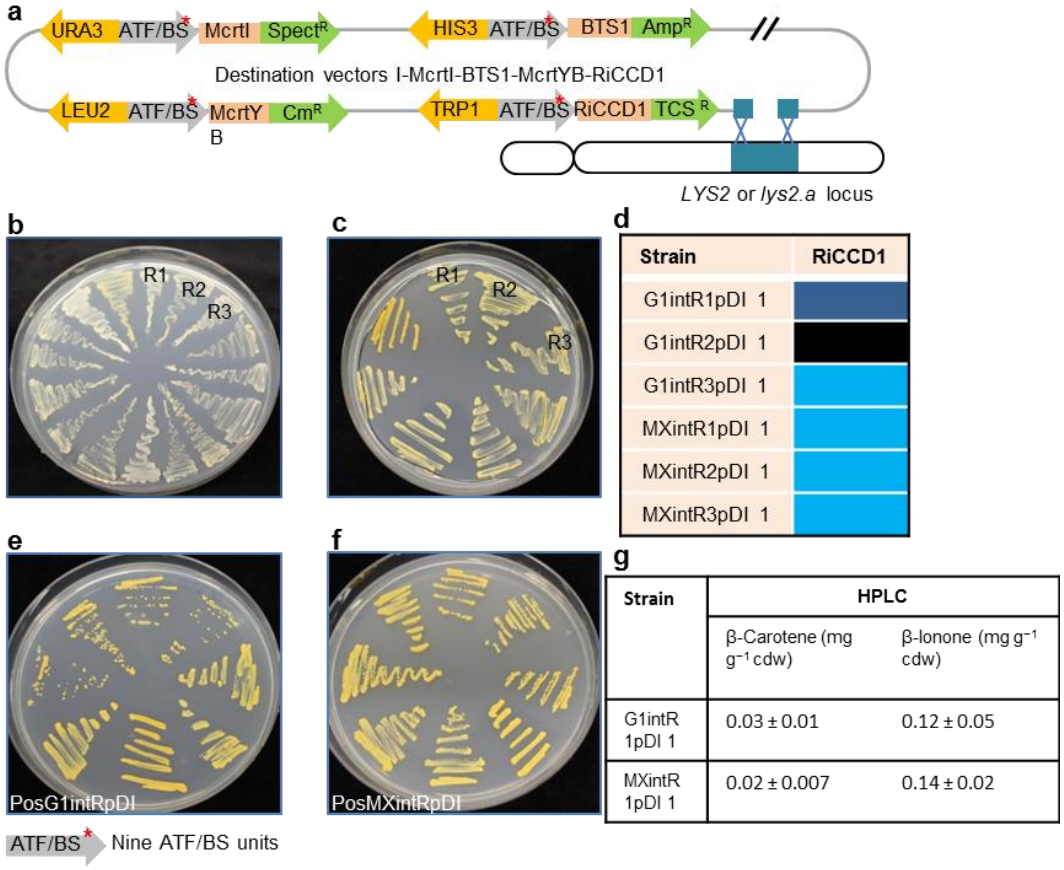

Strain	RiCCD1
G1intR1pDI 1	
G1intR2pDI 1	
G1intR3pDI 1	
MXintR1pDI 1	
MXintR2pDI 1	
MXintR3pDI 1	

Strain	HPLC	
	β-Carotene (mg g^{-1} cdw)	β-Ionone (mg g^{-1} cdw)
G1intR 1pDI 1	0.03 ± 0.01	0.12 ± 0.05
MXintR 1pDI 1	0.02 ± 0.007	0.14 ± 0.02

Supplementary Fig. 17. Single-locus integration of the β-ionone pathway library.

(a) Schematic overview of double strand break-mediated integration of Destination vector I encoding the β-ionone pathway into the yeast genome. The library of the linearized Destination vector I harboring β-ionone modules (β-ionone pathway genes under the control of the nine ATF/BS units) was integrated into the *lys2.a* locus. Selection on SC-Ura/-Leu/-His/-Trp medium allows screening for successfully integrated cassettes. Grey arrows, nine ATF/BS units. For simplicity, the IPTG-inducible promoters upstream of the ATF/BS, terminator fragments, cleavage sites flanking pathway genes and terminators are not included in the figure. **(b)** Integration of the library of modules containing the β-ionone CDSs into strain Gen 0.1 or **(c)** strain IMX672.1. **(d)** Sequencing results for ATF/BS modules upstream of each CDS from three different colonies producing light color in either Gen 0.1 (G1intR1pDI 1, G1intR2pDI 1, and G1intR3pDI 1) or IMX672.1 (MXintR1pDI 1, MXintR2pDI 1, and MXintR3pDI 1) backgrounds. The color code indicating the ATF/BS units assembled upstream of the CDSs is given in Supplementary Fig. 1. **(e)** pIONONE-PTDH3, a Destination vector I containing the β-carotene CDSs under the control of the constitutive yeast *TDH3*

promoter, was integrated into strain Gen 0.1 (PosG1intRpDI) or **(f)** strain IMX672.1 (PosMXintRpDI). **(h)** HPLC analysis of strains MXintR1pDI 1 and G1intR2pDI 1.

Supplementary Notes

Supplementary Note 1

Effect of chromosomal integration site on regulator strength

We sought to confirm the strengths of the nine ATF/BS units employed in COMPASS by testing their transcription output when implemented with other ATF/BS units within a circuit. At Level 1, the nine ATF/BS units were assembled upstream of the *BTS1* CDS units in Acceptor vector A (Supplementary Fig. 11 and Supplementary Data 5). The CDS part was replaced with the *yEGFP* CDS to generate nine ATF/BS-yEGFP modules (see Methods). We then established nine different strains in which ATF/BS-yEGFP modules were integrated into the *XII-5* locus of the yeast genome, while NLS-ATAF1-GAL4AD/2X-McrtI and NLS-DBD_{JUB1}-GAL4AD/2X-McrtYB were integrated into the *X-3* and *XI-3* locus, respectively (Fig. 5a, h, and Supplementary Fig. 2a). Expression of yEGFP under the control of the constitutive yeast *TDH3* promoter was used as a control. These regulators vary in the strength (Supplementary Fig. 2b, Supplementary Data 2a) and noise (Supplementary Fig. 2c, Supplementary Data 2b) properties when driving yEGFP reporter protein expression. These results, together with our previously reported data[1], show that regardless of the genomic integration site (locus *ura3-52* or *XII-5*) of the regulator-reporter cassette the strong ATF/BSs (strains YEGFP1-YEGFP3, Supplementary Fig. 2b, Supplementary Data 2a) lead to higher yEGFP expression and a lower noise than the weak ATF/BSs (strains YEGFP7-YEGFP9, Supplementary Fig. 2b, Supplementary Data 2a) in inducing medium (NLS-JUB1-GAL4AD/2X and NLS-GAL4AD-RAV1/4X are the strongest and weakest regulators, respectively, of our collection). Moreover, all regulators integrated in locus *ura3-52*[1] triggered a higher fluorescence output than regulators integrated in locus *XII-5* (in the β-carotene producing background strain; Student´s *t*-test; *p*-value <0.01; Supplementary Fig. 2b, Supplementary Data 2a). This suggests that the location within the genome significantly affects the expression level of the ATF/BS regulator, probably due to different levels of DNA accessibility. This result gives further weight to a recent study that concluded that the genomic integration position of promoters affects the downstream protein expression level[4].

Supplementary Note 2

Design of vectors

The Level 0 Entry vector X contains the *E. coli pUC19* replication origin, the kanamycin resistance gene *nptII*, the yeast *2μ* replication origin, and a multiple cloning site (*MCS*) flanked by *X0* and *Z0* sequences (Supplementary Fig. 3). A *Pac*I site was placed downstream (right) of *Z0*. Entry vector X is used for two types of cloning reactions: to assemble (i) ATF/BS units with diverse (weak, medium and strong) transcriptional outputs,

and (ii) CDS units (a yeast terminator, CDS, and the promoter of an *E. coli* selection marker) in a single unit (which defines the next level vector).

Acceptor and Destination vectors harbor the *E. coli pUC19* replication origin, the *nptII* kanamycin resistance gene, the yeast *2μ* replication origin, and an *MCS* flanked with *X0* at the upstream (left) side and different *Z* regions (*Z1* to *Z5*) at the downstream (right) sides. The vectors of Set 1 are equipped with functional auxotrophic selection markers placed upstream (left) of the *X0* region and include Destination vector I (*URA3*) (Supplementary Fig. 4a) and Acceptor vectors A (*HIS3*), B (*LEU2*), C (*TRP1*), and D (*LYS2*) (Supplementary Fig. 4b). All vectors of Set 2 harbor dominant selection markers upstream (left) of the *X0* region and include Destination vector II (blp^R, bialaphos resistance gene) (Supplementary Fig. 5a) and Acceptor vector E (*nat1*, nourseothricin resistance gene), F (*ble*, bleomycin resistance gene), G (*neo*, G418 resistance gene), and H (*hph*, hygromycin B resistance gene) (Supplementary Fig. 5b). Moreover, Destination vectors I/II, and Acceptor vectors A/E, B/F, C/G, and D/H harbor the CDSs and terminators of the $Spect^R$, Amp^R, Cmr^R, TCS^R, and Gen^R genes, respectively, downstream (right) of the cloning sites (*MCS* in Acceptor vectors; *p1* in Destination vector I, and *p6* in Destination vector II). In addition, Destination vectors harbor *Y1*, *Y2*, *Y3*, and *Y4* sequences representing homology regions (HRs) to the last 30 bp of the terminators of the Amp^R, Cmr^R, TCS^R, and Gen^R genes, downstream (right) of sites *p2*, *p3*, *p4* and *p5*, respectively (Fig. 2c,e). Destination vector I and II are equipped with the coding DNA sequence (CDS) and terminators of yeast auxotrophic and dominant selection markers, respectively, upstream (left) of the *X1* region (Supplementary Fig. 4b,5b). To integrate the pathways assembled in Destination vectors into the yeast genome, Destination vector I is equipped with *Not*I and *Bam*HI restriction sites allowing plasmid integration into the *URA3* or *LYS2* locus, respectively, while *Pme*I and *Pci*I recognition sequences flanked by homology arms to integrate into *ADE2.a* locus[3] were added to Destination vector II. Additionally, Destination vector I.1 (Supplementary Fig. 6) contains *Pme*I and *Pci*I sequences that are flanked by homology arms for integration into the *LYP1.x* locus[3].

Supplementary Note 3

Growth rate measurements

Methods for growth quantification that are based on colony size or optical density measurements are affected by the growth microenvironment. Methods relying on competitive growth can overcome this issue by measuring relative growth changes of strains competing with each other in a homogeneous environment[4]. To assess whether a growth penalty is associated with the overexpression of artificial transcription factors (ATFs), we employed a high-throughput fitness assay which uses flow cytometry to monitor growth competition of fluorescently labeled yeast strains[5]. To this end, DsRED-expressing (modified) cells and

AcGFP1-expressing (WT) cells (Supplementary Fig. 12a) were co-cultured in non-inducing and inducing medium. The co-cultures were maintained by serial dilution (see Methods). At each time point, samples were removed for analysis by flow cytometry to determine the ratio of DsRED-positive to AcGFP1-positive cells. Rare events that appear to be both DsRED-positive and AcGFP1-positive represent instances in which a modified cell and a wild-type cell are misidentified by the cytometer as the single cell, and we took this into account in our analysis. Because a large number of individual cells (20,000) can be measured by flow cytometry, the assay allows calculating relative growth rates by determining the rate of change of the ratio of modified to WT cells over the course of the competition. We tested Gen 0.1 (Supplementary Fig. 12b, Supplementary Data 8a), CB1 (Supplementary Fig. 12c, Supplementary Data 8b), PCB (Supplementary Fig. 12d, Supplementary Data 8c), IMX672.1 (Supplementary Fig. 12e, Supplementary Data 8d), MXintpDI 1 (Supplementary Fig. 12f, Supplementary Data 8e), and RC1 (Supplementary Fig. 12g, Supplementary Data 8f). In inducing medium, growth rate of strains CB1, MXintpDI 1, and RC1 was reduced by 16%, 7% and 13%, respectively, compared to non-inducing medium. Of note, in non-inducing medium the growth rate of the three strains was virtually the same as in the respective control strains (Gen 0.1 for CB1, and IMX672.1 for MXintpDI 1 and RC1). In contrast, strains CB1, MXintpDI 1 and RC1 showed 15%, 5.5%, and 9.5% reduction in growth rate compared to the corresponding reference strains in inducing medium over the time. Strain PCB, which constitutively expresses the β-carotene pathway, showed 3.6% and 4.5% reduction in growth rate compared to WT in non-inducing and inducing medium, respectively.

Supplementary Note 4

Controllability of IPTG induction utilized in COMPASS

To study IPTG controllability of the expression of plant regulators, the best producers of each of the three COMPASS approaches (see Fig. 5, Supplementary Fig. 13-15), *i.e.*, CB1, G1pDI 1, G1intpDI 1 (β-carotene), RC1, MXR1pDI, and MXR1pDI (β-ionone) and Narion 14 (β-ionone), were plated on both, non-inducing and inducing SC medium with appropriate selection markers (Supplementary Fig. 13a). Strain PCB, which expresses the β-carotene pathway genes from the constitutive yeast *TDH3* promoter, was used as control. Slightly above background expression was observed in non-inducing medium for strains with plant regulators, while expression was high in inducing medium (Supplementary Fig. 13a-c). We observed a yellow color for G1pDI 1 colonies. This is probably due to the formation of an unstable product generated by the enzymes encoded by the episomal plasmid, in agreement with the high standard deviation observed for episomal plasmid-derived β-carotene production (see Fig. 5i). We furthermore investigated β-carotene production of strains PCB1, CB1, G1pDI 1, and G1intpDI 1 in non-inducing and inducing SC medium using the method of

Lian *et al*. (2017)[6] (see Methods). Our results demonstrate that integrating genes into multiple loci of the genome (strain CB1) results in a high-fold induction (factor 8.5) in inducing medium, and 1.7-fold increase compared with strain PCB. Moreover, integration of a whole pathway in a single locus (strain G1intpDI 1) lead to the highest amount of β-carotene accumulation (2.3-fold higher than in the PCB strain) (Supplementary Fig. 13c, Supplementary Data 9).

Supplementary Note 5

Construction of a Destination vector library for controllable β–carotene production

At Level 2, the library of the ATF/BSs-BTS1 modules was integrated into the library of Destination vector I (pDI)-McrtI. Subsequently, the library of ATF/BSs-McrtYB modules was integrated into the library of pDI-McrtI-BTS1 (Fig. 5b), and the combinatorial pDI-McrtI-BTS1-McrtYB library was transformed into either strain Gen 0.1 (Fig. 5d, middle plate) or IMX672.1 (Fig. 5e, middle plate). The selection for successful assemblies was carried out by plating the yeast cells on medium lacking the auxotrophic markers (SC-Ura/-Leu/-His), as the *URA3*, *LEU2*, and *HIS3* genes are encoded on the assembly modules. A variety of colors ranging from light yellow to deep orange were observed in the different colonies obtained from the Gen 0.1 (Fig. 5d, middle plate) and IMX672.1 (Fig. 5e, middle plate) strains. The colony color may result from the presence of several plasmids within a given yeast cell. The plasmids assembled via COMPASS were recovered from the yeast cells and transformed into *E. coli*. The structure of five plasmids was confirmed by sequencing; the sequenced plasmids were then retransformed into Gen 0.1 and IMX672.1 to confirm functional expression of the pathway. Our results showed that combining a weak ATF/BS (NLS-DBD_{JUB1}-GAL4AD/2X) with two strong ATF/BSs (NLS-JUB1-GAL4AD/2X and NLS-ATAF1-GAL4AD/2X) results in superior β-carotene accumulation in both strains (MXpDI 1 and G1pDI 1; Fig. 5i). Moreover, we observed that expressing all three pathway genes from strong ATF/BSs (NLS-ATAF1-GAL4AD/2X upstream of *BTS1* and *McrtYB*; and NLS-JUB1-GAL4AD/2X upstream of *McrtI*) in a Destination vector (pDI 5; Fig. 5i) did not result in high β-carotene accumulation. Moreover, as a control, pCAROTENE-PTDH3 (Destination vector I expressing all three CDSs from the constitutive yeast *TDH3* promoter) was transformed into strains Gen 0.1 (pG1pDI, Fig. 5f, middle plate) and IMX672.1 (pMXpDI, Fig. 5g, middle plate). Although the same promoter controls expression of the three genes, we observed a small variation in the colony colors. In general, (i) we could not reliably probe the stability and robustness of expression of the assembled pathways after retransformation into yeast cells, because the variation in color (light yellow to dark orange) between colonies may result from a variation in the plasmid copy number. (ii) ATF/BS library- or *TDH3* promoter-containing strains established in the Gen 0.1 background (Fig. 5d, f; middle plate) showed better β-carotene accumulation than strains established in strain IMX672.1 (Fig. 5e, h; middle plate). Strains

Gen 0.1 and IMX672.1 with pDI 1 produced colonies with the most intense color. Two colonies, one from strain Gen 0.1 (G1pDI 1) and one from strain IMX672.1 (MXpDI 1) were selected for quantitative determination of β-carotene content by HPLC (Fig. 5i). The HPLC data demonstrated that G1pDI 1 cells accumulated slightly more β-carotene (0.81 ± 0.25 mg g^{-1} cdw) than MXpDI 1 cells (0.61 ± 0.14 mg g^{-1} cdw); this difference, however, was statistically non-significant based on Student´s *t*-test (*p*-value > 0.01), and was probably affected by different plasmid copy numbers in the cells.

Supplementary Note 6

Single-locus integration of a Destination vector I library carrying β-carotene pathway genes

The library of β-carotene pathway genes assembled in Destination vector I from Level 2 was integrated into the *lys2.a* locus of strain Gen 0.1 or IMX672.1 (Fig. 5c, right plate). The selection for integration was carried out on SC-Ura/-Leu/-His plates, as the *URA3*, *LEU2*, and *HIS3* genes are encoded on the β–carotene assembly modules. A spectrum of colors ranging from yellow to deep orange was observed for the different colonies (Gen 0.1 background, Fig. 5d, right plate; IMX672.1 background, Fig. 5e, right plate). ATF/BS modules upstream of each CDS of three deep-orange colonies from each plate (IMX672.1 and Gen 0.1) were amplified by PCR and sequenced. We found that weak to strong, medium, and weak or strong ATF/BS regulators, respectively, drive expression in the *BTS1*, *McrtYB* and *McrtI* modules in the Gen 0.1 background (G1intpDI 1, G1intpDI 2, and G1intpDI 3; Fig. 5h). In the case of strain IMX672.1, *BTS1* expression, recovered from modules of three independent colonies, was controlled by weak or strong ATF/BS regulators, while all *McrtYB* modules contained strong ATF/BS regulators, and *McrtI* expressing modules contained weak or strong ATF/BSs (MXintpDI 1, MXintpDI 2, and MXintpDI 3; Fig. 5h). Moreover, as a control, pCAROTENE-PTDH3 (Destination vector I expressing all three β-carotene biosynthesis genes from the constitutive yeast *TDH3* promoter; see Methods) was integrated into locus *lys2.a* of strains IMX672.1 and Gen 0.1 to generate, respectively, strains PosG1intpDI (Fig. 5f, right plate) and PosMXintpDI (Fig. 5g, right plate). Almost all yeast colonies were similar in color. In general, (i) ATF/BS library- or *TDH3* promoter-containing strains established in Gen 0.1 (Fig. 5d, f; right plate) showed stronger β-carotene accumulation than strains established in the IMX672.1 background (Fig. 5e, g; right plate). We selected two colonies for quantitative determination of β-carotene content by HPLC, one from strain Gen 0.1 (G1intpDI 1; Fig. 5d, right plate), and one from strain IMX672.1 (MXintpDI 1; Fig. 5e; right plate). The HPLC results (Fig. 5i) demonstrated a 3.3-fold higher β-carotene level in the background-optimized strain Gen 0.1 (0.46 ± 0.05 mg g^{-1} cdw) than in the IMX672.1 wild type (0.14 ± 0.05 mg g^{-1} cdw).

Supplementary Note 7

β-Carotene and β-ionone production in control strains

As control for β-carotene and β-ionone quantification in COMPASS-derived strains, we measured their production using HPLC for the background strains, namely IMX672.1 and Gen 0.1. For IMX672.1, we did not detect a considerable amount of β-carotene (0.007 ± 0.001 mg g^{-1} cdw) or β-ionone (0.0008 ± 0.0005 mg g^{-1} cdw). For Gen 0.1, we did not detect a considerable amount of β-carotene (0.015 ± 0.003 mg g^{-1} cdw) or β-ionone (0.0004 ± 0.0002 mg g^{-1} cdw). Beekwilder *et al*. previously reported 0.55 ± 0.005 mg g^{-1} cdw of β-carotene for strain IME167[1], while it produced 0.38 ± 0.08 mg g^{-1} cdw of β-carotene in our experiments (Supplementary Data 10) which is 1.3-fold lower than reported. Moreover, they found that strain RiCCD1[7] produced 0.22 ± 0.06 mg g^{-1} cdw of β-ionone, while it produced 0.042 ± 0.018 mg g^{-1} cdw of β-ionone in our experiments, 5-fold lower than reported.

Supplementary Note 8

Effect of plant-derived ATFs on transcriptional capacity of *cis*-regulatory ATF/BS units

We tested the controls executed by transcription factor NLS-JUB1-EDLLAD-EDLLAD against four copies of JUB1 binding sites inserted upstream of the minimal yeast *CYC1* promoter, with AcGFP1 and DsRED as reporters. To this end, the NLS-JUB1-EDLLAD-EDLLAD-4X-AcGFP1 and NLS-JUB1-EDLLAD-EDLLAD-4X-DsRED modules (see Methods) were integrated into the *XII-5* and *XI-3* sites of the IMX672.1 genome to generate AcGFP1 (AGREEN) and DsRED (BRED) reporter strains, respectively (Supplementary Fig. 14a). To generate strain AB-GREEN-RED, NLS-JUB1-EDLLAD-EDLLAD-4X–AcGFP1 and NLS-JUB1-EDLLAD-EDLLAD-4X–DsRED modules were integrated into the *XII-5* and *XI-3* locus, respectively, of the IMX672.1 genome. The AcGFP1 and DsRED reporter outputs were determined by flow cytometry (Supplementary Fig. 14b, Supplementary Data 11). In strain AB-GREEN-RED, which co-expresses AcGFP1 and DsRED under the control of the NLS-JUB1-EDLLAD-EDLLAD/4X regulator, signal intensity was 34% and 21% lower for AcGFP1 and DsRED, respectively, than in strains expressing the reporters separately.

Supplementary Note 9

Construction of a library for controllable β–carotene and β–ionone production

We established the four-gene pathway required for the production of β-ionone, a downstream product of β-carotene, in the three best β-carotene producers achieved by three approaches. Successful expression of *RiCCD1*, which converts β-carotene to β-ionone, leads to yeast

cells that are less intensely colored than β-carotene-producing cells. At Level 0, we cloned the *RiCCD1* CDS into Entry vector X (Supplementary Data 5). At Level 1, we assembled the library of ATF/BS units and the *RiCCD1* CDS in Acceptor vector C (Supplementary Fig. 7, Supplementary Fig. 11a, and Supplementary Data 6). The nine diverse ATF/BS control modules upstream of the *RiCCD1* CDS were identified by PCR using primers ATF-for and BS-rev, followed by sequencing (Supplementary Fig. 11b). We employed the two yeast strains IMX672.1 and Gen 0.1, and studied the production of β-ionone using methods described in the previous section for analyzing the levels of β-carotene (approach 1, Supplementary Fig. 15; approach 2, Supplementary Fig. 16; and approach 3, Supplementary Fig. 17). Yeast cells expression all for β-ionone CDSs from the *TDH3* promoter were used as positive control. Three weakly colored colonies were chosen to sequence the ATF/BS modules controlling *RiCCD1* expression and one of them was analyzed further by HPLC. We selected the best β-carotene producers in the IMX672.1 and Gen 0.1 backgrounds to introduce the β-ionone library (nine ATF/BS regulators upstream of *RiCCD1*). Moreover, we generated a 9^4-member library of β-ionone producing strains to identify a strain producing β-ionone at the highest possible level (described in the next chapter). We observed that approach 3 (Supplementary Fig. 17), 1 (Supplementary Fig. 15), and 2 (Supplementary Fig. 16) resulted in high, medium and low amounts of β-ionone accumulation, respectively. Surprisingly, in all three cases more β-ionone was produced when pathway genes were assembled in wild-type strain IMX672.1 than in the optimized strain Gen 0.1, confirming that the high level of β-carotene produced in strain Gen 0.1 was not efficiently converted to β-ionone. Overall, our top producer (MXintR1pDI 1; Supplementary Fig. 17g) yielded 3.3-fold more β-ionone than the previously reported strain RiCCD1[7] (0.042 ± 0.018 mg g^{-1} cdw in our experiments; Supplementary Data 10).

Supplementary Note 10

Construction of a library for β-ionone pathway genes by multi-locus integration

The library of ATF/BS-*RiCCD1* CDS modules was integrated into the genomic *ura3-52* locus (Supplementary Fig. 15a) of two strains producing high levels of β-carotene, namely CB1 (Gen 0.1 background, Supplementary Fig. 15b) and CC1 (IMX672.1 background, Supplementary Fig. 15c). The selection for successful assemblies was carried out by plating yeast cells on SC-Ura/-Leu/-His/-Trp/-Lys medium, as the *URA3*, *LEU2*, *HIS3* and *TRP1* genes are encoded by the β–carotene and β–ionone assembly modules and *LYS2* is encoded on the Cas9/sgRNA expression plasmid (see Methods). The enzyme RiCCD1 converts β–carotene to β–ionone whereby yeast colonies lose their deep-orange color. DNA of three light-yellow colonies from each plate (IMX672.1 and Gen 0.1) was used for PCR amplification of ATF/BS modules upstream of the *RiCCD1* CDS, and PCR-amplified

fragments were sequenced (Supplementary Fig. 15d). We found that a weak to medium ATF/BS regulator driving *RiCCD1* expression results in highest β-ionone accumulation in the Gen 0.1 background (RB1, RB2, and RB3; Supplementary Fig. 15b,d), while a medium to strong ATF/BS regulator upstream of *RiCCD1* results in high β-ionone accumulation in the IMX672.1 yeast (RC1, RC2, and RC3; Supplementary Fig. 15c,d). Moreover, three modules expressing *RiCCD1* from the constitutive yeast *TDH3* promoter was integrated in the same locus of Gen 0.1 (PRB, Supplementary Fig. 15e) and IMX672.1 (PRC, Supplementary Fig. 15f). Almost all colonies were similar in color. We selected two colonies for quantitative determination of β-ionone, *i.e.* colony RB1 from strain Gen 0.1 (Supplementary Fig. 15b), and colony RC1 from strain IMX672.1 (Supplementary Fig. 15c). As strains producing high levels of β–carotene (CB1 and CC1) were chosen for genomic integration of the *RiCCD1* CDS (see above), comparing the β–carotene levels in the two host strains with those of the β–ionone-producing strains indicates the extent of β–carotene usage. The HPLC data (Supplementary Fig. 15g) demonstrate a 20- and 14-fold lower β–carotene level in β–ionone expressing strains, RB1 and RC1, than in the backbone strains, CB1 and CC1, respectively, allowing to produce 0.060 ± 0.008 mg g^{-1} cdw and 0.062 ± 0.0001 mg g^{-1} cdw β–ionone in RB1 and RC1, respectively. The highest β-ionone production of 0.22 ± 0.06 mg g^{-1} cdw was previously reported for strain RiCCD1[7], while it produced 0.042 ± 0.018 mg g^{-1} cdw of β-ionone in our experiments (Supplementary Data 10). This represents a 1.5-fold improvement of strain RC1 over RiCCD1[7] (Supplementary Data 10).

Supplementary Note 11

Construction of a Destination vector library for controllable β-ionone production

A library of ATF/BSs-*RiCCD1* modules was assembled in vector pDI 1 (Destination vector I-BTS1-McrtI-McrtYB leading to high β-carotene production) using TAR to generate an episomal plasmid library for β-ionone production (Supplementary Fig. 16a), using strain Gen 0.1 as the host. The generated library was called RpDI library. The selection for clones with correctly assembled pathways was carried out by plating the yeast cells on medium lacking the auxotrophic markers (SC-Ura/-Leu/-His/-Trp); the corresponding genes are encoded by the assembly modules. A variety of colors ranging from light yellow to orange was observed in the different colonies (Supplementary Fig. 16b). As the color of the colonies may result from the presence of different numbers of plasmids within a given yeast cell, plasmids were recovered and transformed into *E. coli*. The structure of three plasmids was confirmed by sequencing; the plasmids were then retransformed into IMX672.1 and Gen 0.1 to confirm functional expression of the pathway. Our results showed that medium to strong ATF/BS regulators resulted in colonies with reduced color formation in both strains (MXR1pDI 1 and G1R1pDI 1; Supplementary Fig. 16c). Moreover, as a control, pIONONE-PTDH3

(Destination vector I expressing all four β-ionone biosynthesis genes from the constitutive yeast *TDH3* promoter; see Methods) was transformed into strain Gen 0.1 (PosG1pDI; Supplementary Fig. 16d). Despite the fact that the same promoter controlled the expression of all four genes, we observed some variation in the colony colors likely due to different plasmid copy numbers. We analyzed the β-ionone content in the G1R1pDI 1 and MXR1pDI 1 strains using HPLC (Supplementary Fig. 16g). The data demonstrate that the amount of β–carotene in strain MXpDI 1 (parental strain with a CEN.PK background) was decreased 20-fold to produce 0.039 ± 0.05 mg g^{-1} cdw β–ionone in strain MXR1pDI 1, while the β–carotene amount in strain G1pDI 1 (parental strain with the optimized biochemical background) was decreased 16-fold to produce 0.036 ± 0.002 mg g^{-1} cdw β–ionone in strain G1R1pDI 1, demonstrating that the high amount of β–carotene cannot be efficiently converted to the downstream product.

Supplementary Note 12

Single-locus integration of a Destination vector I library carrying β-ionone pathway genes

In the next step, the RpDI library containing the nine ATF/BS regulators to control *RiCCD1* expression was integrated into the *lys2.a* locus of strains IMX672.1 and Gen 0.1 (Supplementary Fig. 17a). The selection of successful assemblies was carried out by plating the yeast cells on SC-Ura/-Leu/-His/-Trp medium, as the *URA3*, *LEU2*, *HIS3* and *TRP1* genes are encoded by the assembly modules. A variety of colony colors ranging from light yellow to orange was observed (IMX672.1, Supplementary Fig. 17b; and Gen 0.1, Supplementary Fig. 17c). We checked the sequences of ATF/BS regulators leading to high β-ionone levels (Supplementary Fig. 17d). Our result showed that *RiCCD1* modules need less strong ATF/BS regulators to produce high β-ionone levels in the presence of high precursor levels in the Gen 0.1 background in comparison to IMX672.1. Moreover, as a control, pIONONE-PTDH3 was integrated into the same chromosomal locus of the strains Gen 0.1 (PosG1intRpDI, Fig. Supplementary 17e) and IMX672.1 (PosMXintRpDI, Fig. Supplementary 17f). Almost all colonies grown on solid medium were of similar color. In general, ATF/BS library- or *TDH3* promoter-containing strains established in strain Gen 0.1 (Supplementary Fig. 17b,e) showed better β-carotene consumption (and therefore accumulation of β-ionone) than strains established in strain IMX672.1 (Supplementary Fig. 17c,f). We selected two colonies for quantitative determination of β-ionone content by HPLC (Supplementary Fig. 17g), one from strain Gen 0.1 (G1intR1pDI 1) and one from strain IMX672.1 (MXintR1pDI 1). The HPLC data demonstrate that the amount of β–carotene in strain MXintpDI 1 (wild-type background) was decreased 7-fold to produce 0.14 ± 0.02 mg g^{-1} cdw β-ionone in MXintR1pDI 1, while the β–carotene amount in strain G1intpDI 1 (optimized

background) was decreased 15-fold to produce 0.12 ± 0.06 mg g^{-1} cdw β–ionone in G1intR1pDI 1. This result is in accordance with our previous observation that a high level of precursor (β-carotene) does not necessarily result in high amounts of the downstream metabolite (β-ionone). In conclusion, we generated a β-ionone producing yeast strain, named MXintR1pDI 1, which accumulates 3.3-fold more β-ionone than the previously reported strain RiCCD1[7] (Supplementary Data 10).

Supplementary Methods

Combinatorial assembly of plant-derived ATFs and BSs in Entry Vector X

1. Expression plasmids[1] containing the coding sequences (CDSs) of plant-derived artificial transcription factors (ATFs) are mixed in equimolar ratio and DNA fragments harboring the IPTG-inducible *GAL1* promoter, the CDSs of plant derived ATFs and the *CYC1* terminator ($Pro_{mGAL1\text{-}LacI}$-ATF-Ter_{CYC1}) are amplified by multiplex-PCR using primers ATF_for and ATF_rev.
    - Primer ATF_for overlaps with the *X0* region.
    - Primer ATF_rev overlaps with primer BS_for.
2. Reporter plasmids[1] containing the binding sites (BSs) of ATFs are mixed in equimolar ratios and DNA fragments harboring one, two or four copies of the binding sites ($Pro_{CYC1min\text{-}BS}$) are obtained by multiplex-PCR using primers BS_for and BS_rev.
    - Primer BS-rev overlaps with the *Z0* region, representing the last 30 bp of the minimal *CYC1* promoter.
3. Entry vector X is digested at the multiple cloning site (*MCS)*.
4. Multiplex-PCR-amplified ATFs, their corresponding BS fragments, and linearized Entry vector X are mixed at a molar ratio of 2:10:1 to perform NEBuilder HiFi DNA assembly in a single reaction tube.
5. The cloning reaction is transformed into *E. coli* and plated onto LB agar media containing Kanamycin (50 µg/ml).
6. Colony-PCR is performed using primers EXSEQ-for/EXSEQ-rev and primers PROSEQ-for/PROSEQ-rev to identify the respective combinations of ATFs and BSs, respectively.
7. Desired colonies are inoculated into LB liquid medium containing Kanamycin (50 µg/ml) for sequencing to identify the respective combinations of ATFs and BSs.

- Sequences *X0* and *Z0* are explained in the footnote to Supplementary Data 4.

Design of primers for the assembly of CDS units in Entry vector X

1. Forward primer for PCR amplification of CDSs:
    - The upstream (left) homology region (HR) of the linearized vector (called *Z0*) defines the upstream (left) HR of the primer.
    - A rare restriction enzyme (RE) cleavage site is introduced upstream the gene´s translation start codon that is compatible to the next level vectors (Destination vectors I/II: *Asi*SI; Acceptor vectors A/E: *I-Ceu*I, B/F: *I-Sce*I, C/G: *PI-Psp*I, and E/F: *PI-Sce*I/*Asc*I). For optional possibilities see end of this section.
2. Reverse primer for PCR amplification of CDSs:

- The downstream (right) HR of the primer is defined based on the upstream region of the chosen yeast terminator.

3. Forward primer for PCR amplification of the terminator:
   - The primer is defined based on the upstream region of the chosen yeast terminator.
4. Reverse primer for PCR amplification of the terminator:
   - The same rare RE recognition site as above is introduced downstream of the terminator. For optional possibilities see end of this section.
5. Forward primer for PCR amplification of the promoter of the *E. coli* selection marker:
   - The upstream (left) HR of the primer overlaps with the yeast terminator.
6. Reverse primer for PCR amplification of the promoter of the *E. coli* selection marker:
   - The downstream (right) HR of the primer overlaps with the *Y0* region of the vector and introduces Z (*Z1, Z2, Z3, Z4,* or *Z5)* sequences, representing the first 30 bp of the CDSs of the *E. coli* selection markers encoded by the next-level vectors.

Sequences *Y0* and *Z0 - Z5* are explained in the footnote to Supplementary Data 4.

Optional: Introducing the RE sites in the primer sequences allows replacement of the CDS(s) and yeast terminator(s) with other CDS(s) and terminator(s) at a later stage when alternative COMPASS vectors are needed.

Combinatorial assembly of ATF/BS units and genes in Level 1 vectors

1. Nine Entry vectors X-ATF/BS containing the CDSs of plant-derived ATFs and BSs are mixed in equimolar ratio. The ATF/BS units ($Pro_{mGAL1\text{-}LacI}$-ATF-Ter_{CYC1}-$Pro_{CYC1min\text{-}BS}$) are PCR-amplified using primers X0_for/Z0_rev.
   - Primer X0_for overlaps with the *X0* region in the vectors.
   - Primer Z0_rev overlaps with the last 30 bp of the *CYC1* promoter.
2. Five Entry vectors X-CDS are mixed in equimolar ratio. The CDS units (CDS-Ter_{yeast}-$Pro_{E.coli}$) are PCR-amplified using primers Z0_for, Z1_rev, Z2_rev, Z3_rev, Z4_rev and Z5_rev at a molar ratio of 5:1:1:1:1:1.
   - Primer Z0_for, representing the last 30 bp of the *CYC1* promoter, overlaps with primer Z0_rev.
   - Primers Z1_rev, Z2_rev, Z3_rev, Z4_rev, and Z5_rev overlap with the *Z1* (Destination vector I), *Z2* (Acceptor vectors A), *Z3* (Acceptor vectors B), Z4 (Acceptor vectors C), and *Z5* (Acceptor vectors D), representing the first 30 bp of the *CDSs* of the spectinomycin (*$Spect^R$*), ampicillin (*Amp^R*), chloramphenicol (*Cm^R*), triclosan (*TCS^R*), or gentamicin (*Gen^R*) resistance gene, respectively.
3. The *Sal*I/*Eco*RI-digested Destination vector I and *Fse*I/*Asc*I-digested Acceptor vectors A, B, C, and D are mixed in equimolar ratio.

4. Multiplex PCR-amplified (nine) ATF/BS units, multiplex PCR-amplified (five) CDS units, and (five) linearized vectors are mixed at a molar ratio of 2:2:1 to perform NEBuilder HiFi DNA assembly in a single reaction tube.
5. The reaction cocktail is transformed into *E. coli*.
6. The *E. coli* cells are plated onto LB agar media containing either spectinomycin (50 µg/ml), ampicillin (50 µg/ml), chloramphenicol (25 µg/ml), triclosan (14.5 µg/ml), or gentamicin (50 µg/ml) to generate Destination vectors I-(ATF/BS-)CDS1, and Acceptor vectors A-(ATF/BS-)CDS2, B-(ATF/BS-)CDS3, C-(ATF/BS-)CDS4, and D-(ATF/BS-)CDS5.
7. Colony-PCR is performed using primers ATF-for and BS-rev.
8. Desired colonies are inoculated into LB liquid medium containing appropriate antibiotic (see step 6) for sequencing to identify the respective combinations of ATFs and BSs.

- Sequences *X0* and *Z0 - Z5* are explained in the footnote to Supplementary Data 4.

Design of primers for combinatorial assembly of gene units in Level 1 vectors

1. Forward primer for PCR amplification of CDSs:
   The upstream (left) HR of the linearized vector (called *X0*) defines the upstream (left) HR of the primer.
   A rare RE cleavage site is introduced upstream the gene´s translation start codon that is compatible to the vectors (Destination vectors I/II: *Asi*SI, Acceptor vectors A/E: *I-Ceu*I, B/F: *I-Sce*I, C/G: *PI-Psp*I, and E/F: *PI-Sce*I/*AscI*). For optional possibilities see end of this section.
2. Reverse primer for PCR amplification of CDSs:
   The downstream (right) HR of the primer is defined based on the upstream region of the chosen yeast terminator.
3. Forward primer for PCR amplification of the terminator:
   The primer is defined based on the upstream region of the chosen yeast terminator.
4. Reverse primer for PCR amplification of the terminator:
   The same rare RE recognition site as above is introduced downstream of the terminator. For optional possibilities see end of this section.
5. Forward primer for PCR amplification of the promoter of the *E. coli* selection marker:
   The upstream HR of the primer overlaps with the yeast terminator.
6. Reverse primer for PCR amplification of the promoter of the *E. coli* selection marker:
   The downstream (right) HR of the primer overlaps with *Z1* (Destination vector I/I.1/II), *Z2* (Acceptor vectors A/E), *Z3* (Acceptor vectors B/F), *Z4* (Acceptor vectors C/G), and *Z5* (Acceptor vectors D/H), representing the first 30 bp of the CDSs of $Spect^R$, Amp^R, Cm^R, TCS^R, or Gen^R, respectively.

Sequences *X0* and *Z0 - Z5* are explained in the footnote to Supplementary Data 4.
Optional: Introducing the RE sites in the primer sequences allows replacement of the CDS(s) and yeast terminator(s) with other CDS(s) and terminator(s) at a later stage when alternative COMPASS vectors are needed.

Combinatorial assembly of pathways in Destination vector I (or I.1) at Level 2

1. Destination vectors I-(ATF/BS-)CDS1 are mixed at equimolar molar and digested with *I-Ceu*I or/and *Sbf*I (without gel-purification).
2. The library of Acceptor vectors A-(ATF/BS-)CDS2 is mixed at equimolar molar and used for multiplex PCR amplification of ATF/BS-CDS2 modules (Pro_{HIS3}-Pro_{mGAL1}-*ATF/BS-CDS2*-Ter_{yeast}-Pro_{AmpR}-Amp^{R}-Ter_{AmpR}) using primers X0_for and Y1_rev.
3. Using TAR, the PCR products are assembled in the library of Destination vectors I-(ATF/BS-)CDS1 digested with *I-Ceu*I or/and *Sbf*I to generate a library of Destination vectors I-(ATF/BS-)CDS1-(ATF/BS-)CDS2.
4. Yeast cells are plated on SC-Ura/-His medium.
5. After 4 d, cells are scraped from the plates and the plasmid library is extracted.
6. The recovered plasmid library is transformed into *E. coli*, and cells are grown on LB agar plates containing ampicillin (50 µg/ml).
7. *E. coli* cells are scraped from the plates and the plasmid library is extracted.
8. The library of Acceptor vectors B-(ATF/BS-)CDS3 is used for multiplex PCR amplification of ATF/BS-CDS3 modules (Pro_{LEU2}-Pro_{mGAL1}-*ATF/BS-CDS3*-Ter_{yeast}-Pro_{CmR}-Cm^{R}-Ter_{CmR}) using primers X0_for and Y2_rev.
9. Using TAR, the PCR products are assembled in the library of Destination vectors-(ATF/BS-)CDS1-(ATF/BS-)CDS2 digested with *I-Sce*I or/and *Fse*I (without gel-purification) to generate a library of Destination vectors I-(ATF/BS-)CDS1-(ATF/BS-)CDS2-(ATF/BS-)CDS3.
10. Yeast cells are plated on SC-Ura/-His/-Leu medium.
11. After 4 d, cells are scraped from the plates and the plasmid library is extracted.
12. The recovered plasmid library is transformed into *E. coli*, and cells are grown on LB agar plates containing chloramphenicol (25 µg/ml).
13. *E. coli* cells are scraped from the plates and the plasmid library is extracted.
14. The library of Acceptor vectors C-(ATF/BS-)CDS4 is used for multiplex PCR amplification of ATF/BS-CDS4 modules (Pro_{TRP1}-Pro_{mGAL1}-*ATF/BS-CDS4*-Ter_{yeast}-Pro_{TCSR}-TCS^{R}-Ter_{TCSR}) using primers X0_for and Y3_rev.
15. Using TAR, the PCR products are assembled in the library of Destination vectors-(ATF/BS-)CDS1-(ATF/BS-)CDS2-(ATF/BS-)CDS3 with *PI-Psp*I or/and *Sfi*I digested

(without gel-purification) to generate a library of Destination vectors I-(ATF/BS-)CDS1-(ATF/BS-)CDS2-(ATF/BS-)CDS3-(ATF/BS-)CDS4.

16. Yeast cells were plated on SC-Ura/-His/-Leu/-Trp medium.
17. After 4 d, cells are scraped from the plates and the plasmid library is extracted.
18. The recovered plasmid library is transformed into *E. coli*, and cells are grown on LB agar plates containing triclosan (14.5 µg/ml).
19. *E. coli* cells are scraped from the plates and the plasmid library is extracted.
20. The library of Acceptor vectors D-(ATF/BS-)CDS5 is used for multiplex PCR amplification of ATF/BS-CDS5 modules (Pro_{LYS2}-Pro_{mGAL1}-*ATF/BS-CDS5*-Ter_{yeast}-Pro_{GenR}-Gen^R-Ter_{GenR}) using primers X0_for and Y4_rev.
21. Using TAR, the PCR products are assembled in the library of Destination vectors-(ATF/BS-)CDS1-(ATF/BS-)CDS2-(ATF/BS-)CDS3-(ATF/BS-)CDS4 digested with *AscI* (without gel-purification) to generate a library of Destination vectors I-(ATF/BS-)CDS1-(ATF/BS-)CDS2-(ATF/BS-)CDS3-(ATF/BS-)CDS4-(ATF/BS-)CDS5.
22. Yeast cells are plated on SC-Ura/-His/-Leu/-Trp/-Lys medium.
23. After 4 d, cells are scraped from the plates and plasmid library is extracted.
24. The recovered plasmid library is transformed into *E. coli*, and cells are grown on LB agar plates containing gentamicin (50 µg/ml).
25. *E. coli* cells are scraped from the plates and the plasmid library is extracted.

- Sequence *X0* is explained in the footnote to Supplementary Data 4.
- Sequences *Y1, Y2, Y3,* and *Y4* represent to the last 30 bp of the terminator sequences of the Amp^R, Cm^R, TCS^R, or Gen^R gene, respectively.

Supplementary References

1. Naseri, G., Balazadeh, S., Machens, F., Kamranfar, I., Messerschmidt, K. & Mueller-Roeber, B. Plant-derived transcription factors for orthologous regulation of gene expression in the yeast *Saccharomyces cerevisiae*. *ACS Synth Biol* **6,** 1742-1756 (2017).
2. Bao Z*, et al.* Homology-integrated CRISPR-Cas (HI-CRISPR) system for one-step multigene disruption in *Saccharomyces cerevisiae*. *ACS Synth Biol* **4**, 585-594 (2015).
3. Lian J, HamediRad M, Hu S, Zhao H. Combinatorial metabolic engineering using an orthogonal tri-functional CRISPR system. *Nat Commun* **8**, 1688 (2017).
4. Redden H, Alper HS. The development and characterization of synthetic minimal yeast promoters. *Nat Commun* **6**, 7810 (2015).
5. Breslow DK*, et al.* A comprehensive strategy enabling high-resolution functional analysis of the yeast genome. *Nat Methods* **5**, 711-718 (2008).
6. Lian J, HamediRad M, Hu S, Zhao H. Combinatorial metabolic engineering using an orthogonal tri-functional CRISPR system. *Nat Commun* **8**, 1688 (2017).
7. Beekwilder J*, et al.* Polycistronic expression of a beta-carotene biosynthetic pathway in *Saccharomyces cerevisiae* coupled to beta-ionone production. *J Biotechnol* **192 Pt B**, 383-392 (2014).
