# Supplementary material for: COMPASS for rapid combinatorial optimization of biochemical pathways based on artificial transcription factors
Source: Nat Commun. 2019 Jun 13;10:2615. doi: 10.1038/s41467-019-10224-x (PMC6565718; doi:10.1038/s41467-019-10224-x)
Supplement: Supplementary file 17 — Reporting Summary [file 41467_2019_10224_MOESM17_ESM.pdf]

nature research

Corresponding author(s): Bernd Mueller-Roeber

Last updated by author(s): Apr 19, 2019

# Reporting Summary

Nature Research wishes to improve the reproducibility of the work that we publish. This form provides structure for consistency and transparency in reporting. For further information on Nature Research policies, see Authors & Referees and the Editorial Policy Checklist.

## Statistics

For all statistical analyses, confirm that the following items are present in the figure legend, table legend, main text, or Methods section.

| n/a | Confirmed | |
|---|---|---|
| ☐ | ☒ | The exact sample size (*n*) for each experimental group/condition, given as a discrete number and unit of measurement |
| ☐ | ☒ | A statement on whether measurements were taken from distinct samples or whether the same sample was measured repeatedly |
| ☒ | ☐ | The statistical test(s) used AND whether they are one- or two-sided<br>*Only common tests should be described solely by name; describe more complex techniques in the Methods section.* |
| ☒ | ☐ | A description of all covariates tested |
| ☒ | ☐ | A description of any assumptions or corrections, such as tests of normality and adjustment for multiple comparisons |
| ☒ | ☐ | A full description of the statistical parameters including central tendency (e.g. means) or other basic estimates (e.g. regression coefficient) AND variation (e.g. standard deviation) or associated estimates of uncertainty (e.g. confidence intervals) |
| ☒ | ☐ | For null hypothesis testing, the test statistic (e.g. *F*, *t*, *r*) with confidence intervals, effect sizes, degrees of freedom and *P* value noted<br>*Give P values as exact values whenever suitable.* |
| ☒ | ☐ | For Bayesian analysis, information on the choice of priors and Markov chain Monte Carlo settings |
| ☒ | ☐ | For hierarchical and complex designs, identification of the appropriate level for tests and full reporting of outcomes |
| ☒ | ☐ | Estimates of effect sizes (e.g. Cohen's *d*, Pearson's *r*), indicating how they were calculated |

*Our web collection on statistics for biologists contains articles on many of the points above.*

## Software and code

Policy information about availability of computer code

| | |
|---|---|
| Data collection | -HPLC analyses (performed by AppliChrom, www.applichrom.de)<br>-BD FACSCalibur Flow Cytometer (BD Biosciences) |
| Data analysis | Flowing Software version 2.5.1 (Turku Centre for Biotechnology, http://flowingsoftware.btk.fi) |

For manuscripts utilizing custom algorithms or software that are central to the research but not yet described in published literature, software must be made available to editors/reviewers. We strongly encourage code deposition in a community repository (e.g. GitHub). See the Nature Research guidelines for submitting code & software for further information.

## Data

Policy information about availability of data

All manuscripts must include a data availability statement. This statement should provide the following information, where applicable:
- Accession codes, unique identifiers, or web links for publicly available datasets
- A list of figures that have associated raw data
- A description of any restrictions on data availability

- Flow cytometry and HPLC data, supplementary notes and figures, gene accession numbers and sequences, plasmid and primer sequences are available in the Supplementary Information.
- Step-by-step experimental protocols for all combinatorial assemblies are included in the Supplementary Information.
- The 'Reporting Summary' for this article is available as a Supplementary Information file.
- All other relevant data and materials are available from the corresponding author on request.

# Field-specific reporting

Please select the one below that is the best fit for your research. If you are not sure, read the appropriate sections before making your selection.

☒ Life sciences ☐ Behavioural & social sciences ☐ Ecological, evolutionary & environmental sciences

For a reference copy of the document with all sections, see nature.com/documents/nr-reporting-summary-flat.pdf

# Life sciences study design

All studies must disclose on these points even when the disclosure is negative.

**Sample size**

Diversity in COMPASS library is defined by the number of regulators (Y) and open reading frames (N).
- Narion library: 0.0000025% of the theoretical complexity of the library was selected to study the optimization capacity of COMPASS.
- Beta-carotene producing libraries achieved using any of the COMPASS approaches (detailed information available in Results): the three best producers (intensive orange color of colonies) of the library with a theoretical complexity of 729 members were selected to check COMPASS optimization capacity.
- Beta-ionone producing library achieved using any of the COMPASS approaches (detailed information available in Results): the three best producers (with less orange color) of the library with a theoretical complexity of nine members were selected to check COMPASS optimization capacity.

**Data exclusions**

No data were excluded from the analyses.

**Replication**

- Gene sequencing was not replicated.
- The experimental data for HPLC, flow cytometry, beta-carotene quantification (using the protocol described by Lia et al., 2017), and growth measurements were replicated and replicate experiments were successfully performed.

**Randomization**

- All constructs at Level 1 and Level 2 were generated randomly.
- Nine different Level 1 constructs for each gene of the beta-carotene and beta-ionone pathways were identified by sequencing plasmid inserts of 26 to 29 randomly selected individual E. coli colonies.
- Yeast Narion strains were selected at random to study the versatility and optimization capacity of COMPASS.

**Blinding**

- Investigators were not blinded to choose beta-carotene and beta-ionone strains for quantifying beta-carotene and beta-ionone production (pre-screening for better producers was performed based on color type and intensity).
- Investigators were blinded to choose Narion strains for quantifying naringenin and beta-ionone production.
- Investigators were blinded regarding sequences of COMPASS Level 0 units and Level 1 modules for naringenin pathway genes (constructs were not sequenced).
- Investigators were blinded regarding selected colonies for growth measurements.

# Reporting for specific materials, systems and methods

We require information from authors about some types of materials, experimental systems and methods used in many studies. Here, indicate whether each material, system or method listed is relevant to your study. If you are not sure if a list item applies to your research, read the appropriate section before selecting a response.

## Materials & experimental systems

| n/a | Involved in the study | |
|---|---|---|
| ☒ | ☐ | Antibodies |
| ☒ | ☐ | Eukaryotic cell lines |
| ☒ | ☐ | Palaeontology |
| ☒ | ☐ | Animals and other organisms |
| ☒ | ☐ | Human research participants |
| ☒ | ☐ | Clinical data |

## Methods

| n/a | Involved in the study | |
|---|---|---|
| ☒ | ☐ | ChIP-seq |
| ☐ | ☒ | Flow cytometry |
| ☒ | ☐ | MRI-based neuroimaging |

# Flow Cytometry

## Plots

Confirm that:

- [ ] The axis labels state the marker and fluorochrome used (e.g. CD4-FITC).
- [ ] The axis scales are clearly visible. Include numbers along axes only for bottom left plot of group (a 'group' is an analysis of identical markers).
- [ ] All plots are contour plots with outliers or pseudocolor plots.
- [ ] A numerical value for number of cells or percentage (with statistics) is provided.

## Methodology

| | |
|---|---|
| Sample preparation | Sample preparation is described in Online Methods. |
| Instrument | BD FACSCalibur Flow Cytometer (BD Biosciences) |
| Software | Flowing Software version 2.5.1 (Turku Centre for Biotechnology, http://flowingsoftware.btk.fi) |
| Cell population abundance | For each culture, 20,000 events were collected.<br>For the growth measurement using flow cytometry (Supplementary Figure 12):<br>(i) The population of CB1 cells producing beta-carotene (before integration of the DsRED expression cassette into the genome) was used as a control to check the background by flow cytometry. The data showed no presence of beta-carotene when using the DsRED pass filter.<br>(ii) To mix the modified and wild-type cell populations, ratios 9:1, 8:2, and 6:4 were tested for the cells Gen 01_DsRED and Gen 0.1_AcGFP1. The ratio 8:2 was selected as an optimal ratio to mix the modified and the wild-type cells.<br>(iii) Cell populations producing DsRED or AcGFP1 in each mixed sample were used for analysis. |
| Gating strategy | The total cell population in a sample was gated in a bivariate dot plot of FSC and SSC. The instrument settings were in logarithmic mode: FSC-H and SSC-H. |

- [ ] Tick this box to confirm that a figure exemplifying the gating strategy is provided in the Supplementary Information.
