# Supplementary material for: COMPASS for rapid combinatorial optimization of biochemical pathways based on artificial transcription factors
Source: Nat Commun. 2019 Jun 13;10:2615. doi: 10.1038/s41467-019-10224-x (PMC6565718; doi:10.1038/s41467-019-10224-x)
Supplement: Supplementary file 18 — Description of Additional Supplementary Files [file 41467_2019_10224_MOESM18_ESM.pdf]

**Title:** Supplementary Data 1.
**Description:** Comparison of various combinatorial cloning methodologies.

**Title:** Supplementary Data 2.
**Description:** Position effects of plant-derived regulators.

**Title:** Supplementary Data 3.
**Description:** List of COMPASS vectors.

**Title:** Supplementary Data 4.
**Description:** PCR fragments used for the cloning of parts into Entry vector X.

**Title:** Supplementary Data 5.
**Description:** Parts of genes assembled in Entry vector X.

**Title:** Supplementary Data 6.
**Description:** Parts of genes assembled in entry vectors.

**Title:** Supplementary Data 7.
**Description:** List of *Saccharomyces cerevisiae* strains used in this study.

**Title:** Supplementary Data 8.
**Description:** High-precision flow cytometry for growth analysis.

**Title:** Supplementary Data 9.
**Description:** Controllability of plant-derived regulators in COMPASS strains.

**Title:** Supplementary Data 10.
**Description:** HPLC results for control strains.

**Title:** Supplementary Data 11.
**Description:** Transcriptional output of the NLS-JUB1-EDLLAD-EDLLAD-derived ATF.

**Title:** Supplementary Data 12.
**Description:** Primers used for sequencing the library integrated into the genome.

**Title:** Supplementary Data 13.
**Description:** Diversity of β-ionone and NG production from a randomized ATF/BS library.

**Title:** Supplementary Data 14.
**Description:** Sequences of vectors used in this study.

**Title:** Supplementary Data 15.
**Description:** Sequences of primers used in this study.
